# Nucleocapsid protein captures DDX5 and RNMT facilitating viral RNA synthesis and viral protein translation for coronavirus replication

**DOI:** 10.1128/mbio.02717-25

**Published:** 2026-01-26

**Authors:** Yuchang Liu, Ning Kong, Xinyu Yang, Wenzhen Qin, Yahe Wang, Chen Wang, He Sun, Jiarui Wang, Ao Gao, Dongfang Zheng, Wu Tong, Hai Yu, Hao Zheng, Guangzhi Tong, Tongling Shan

**Affiliations:** 1 Shanghai Veterinary Research Institute, Chinese Academy of Agricultural Sciences 118161 , Shanghai, Shanghai, China; 2 Jiangsu Co-Innovation Center for the Prevention and Control of Important Animal Infectious Disease and Zoonose, Yangzhou University 38043, Yangzhou, Jiangsu, China; Johns Hopkins University Bloomberg School of Public Health, Baltimore, Maryland, USA

**Keywords:** coronaviruses, RNA-binding proteins, virus-host interaction, RNA synthesis, protein translation

## Abstract

Coronaviruses (CoVs) hijack host RNA-binding proteins (RBPs) to facilitate their replication, but the viral proteins and host RBDs that participate in the synthesis of viral RNA and protein are unclear. In this study, we revealed that DEAD-box helicase (DDX5) and staphylococcal nuclease domain-containing protein (SND1) facilitate viral RNA synthesis and that RNA guanine-7 methyltransferase (RNMT) enhances viral protein translation to promote viral replication via coronaviral subgenomic RNA-protein interactomes. DDX5 and SND1 positively regulate PEDV replication by promoting viral RNA synthesis via the binding of DDX5 to positive-sense viral RNA, whereas SND1 specifically detects negative-sense viral RNA. The interaction of DDX5/SND1 and N/nsp9/nsp12 promotes the formation of replication-transcription complexes for viral RNA synthesis to facilitate viral replication. We found that RNMT captures the host protein translation system to cyclize viral mRNA to assist in viral protein translation to promote viral replication. We also found that DDX5 broadly interacts with the N protein of CoVs and promotes the RNA synthesis of bovine coronavirus and porcine delta-coronavirus to promote viral replication. These results indicate that CoVs use host proteins to assist in the synthesis of viral RNA and protein to facilitate viral replication.

**IMPORTANCE:**

The synthesis of viral RNA and proteins is a crucial process in the life cycle of CoVs. Our observations indicate that DDX5 and SND1 facilitate the assembly of viral replication-transcription complexes and enhance viral RNA synthesis, with DDX5 binding to positive-sense RNA and SND1 binding to negative-sense RNA. Meanwhile, RNMT promotes viral protein translation by hijacking the host translation machinery and mediating the circularization of viral mRNA. These findings offer new insights into the mechanisms through which coronaviruses exploit both viral and host proteins to synthesize viral RNA and proteins.

## INTRODUCTION

Coronaviruses (CoVs) that threaten humans and livestock are classified into four genera, including *Alpha*, *Beta*, *Gamma*, and *Delta* (1, 2), whose genomes are positive-sense single-stranded RNAs (+ssRNAs) containing 5′ capped and 3′-polyA that directly utilize host protein translation systems including RNA-binding proteins (RBPs) and ribosomes, together with translation factors, during the onset of viral genome translation to synthesize the replicase polyproteins by ORF1a and ORF1b (3). The replicase polyproteins are cleaved into 16 nonstructural proteins (nsps) containing enzymes to assemble the replication and transcription complexes (RTCs) required for synthesizing the negative-strand template, positive-strand genomic RNA, and subgenomic RNAs (sgRNAs) (4, 5). The subgenomic mRNAs (sgmRNAs) of CoVs are synthesized by discontinuous transcription via the template-switching mechanism to fuse the genomic 5′ leader to the viral sgRNA to produce four structural proteins (S, E, M, and N) and several species-specific accessory proteins by capturing host protein translation systems (6–9). Along with the emergence of CoVs, severe acute respiratory syndrome coronavirus (SARS-CoV) (10), Middle East respiratory syndrome coronavirus (MERS-CoV) (10), and severe acute respiratory syndrome coronavirus 2 (SARS-CoV-2) threaten human health and society (11); the emergence of CoVs, including porcine deltacoronavirus (PDCoV) and swine acute diarrhea syndrome coronavirus (SADS-CoV) (12, 13), and the reappearance of porcine enteric diarrhea virus (PEDV) have severely harmed the pig industry (14). Especially, PEDV has caused considerable economic losses in the pig farming industry (15–19). A better understanding of the host and viral proteins that are essential for viral RNA synthesis and viral protein translation for coronavirus replication is needed to facilitate the discovery of antiviral targets (5, 6).

DEAD-box polypeptide 5 (DDX5) is a member of the DEAD box family of RNA helicases that participate in various cellular processes, promoting interactions with many other factors, which play roles in altering RNA structures, the coregulation of transcription, the regulation of splicing, the nuclear export of mRNAs, and the processing of small noncoding RNAs (20, 21). DDX5 can be shuttled between the nucleus and the cytoplasm to interact with viral proteins to promote the replication of SARS-CoV by interacting with nsp13 (22), porcine reproductive and respiratory syndrome virus (PRRSV) by interacting with nsp9 (23), and influenza virus by colocalizing with viral NPs (24). RTC activity requires cellular host factors to promote viral RNA synthesis for the replication of viruses. The components of the core viral RNA synthesis machinery are well-characterized (5), but the regulatory roles of host proteins in CoV RNA biosynthesis remain poorly understood, and whether DDX5 is a key host factor necessary for the RTC for CoV replication is unknown.

The RNA guanine-7 methyltransferase (RNMT), known as mRNA 5′-cap (guanine-N7-)-methyltransferase (guanine-N7 MTase) and RNA binding protein (RBP), adds a methyl group to GpppN, forming the mature cap (m7GpppN) after RNA triphosphatase (TPase) removes the γ-phosphate and RNA guanylyltransferase (GTase), which transfers a GMP group to the 5′ diphosphate (25–27). The caps of the viral genome and sgRNA play important roles in allowing viruses to evade host restriction and assisting in viral protein translation (28, 29). The nsp14 of CoV contains an N7-MTase domain in the C-terminus, which catalyzes mRNA capping action, and an exonuclease (ExoN) domain in the N-terminus, which is required for the activity of N7-MTase and interacts with nsp10 recruited by nsp9 and nsp12, forming a capping complex to add a cap at the 5′ end of pre-mRNA (5, 30–32). While rescuing CoV by reverse genetic systems in cells, the viral genome expressed by eukaryotic plasmids needs to be capped by host proteins because the nsps of CoVs, including nsp9/10/12/14, are lacking (33–35). RNMT, a guanine-N7 MTase that includes TPase and GTase, might participate in capping the transcription of the CoV genome from eukaryotic plasmids to rescue the CoV by reverse genetic systems. In the last step of RNA capping, RNMT adds a methyl group to GpppN, forming the mature cap (m7GpppN), and capped RNAs that bind RNMT are readily translated; however, whether RNMT is involved in mRNA translation is not known.

The CoV proteins that hijack host RBDs to facilitate viral RNA synthesis and viral protein translation to facilitate the replication of CoVs need to be identified (11). In this study, we analyzed the interactomes of CoV RNA and host proteins across *α*-, *β*-, and *δ*-CoVs using RNA pull-down proteomics and found that DDX5, SND1, and RNMT, as host factors that bind to pan-coronavirus RNA, significantly promoted the replication of PEDV. We found that the N protein of PEDV recruited DDX5, SND1, and nsp12/9 to drive RTC complex assembly to increase viral RNA synthesis by binding viral RNA and capturing RNMT, which interacted with host protein translation systems to facilitate the translation of viral mRNA to promote PEDV replication. We also revealed that the interaction of DDX5 with the pan-N protein of CoVs facilitates viral replication by assisting in the RNA synthesis of bovine coronavirus and porcine delta-coronavirus.

## RESULTS

### Proteinome interactions with coronaviral RNA

**Fig 1:**
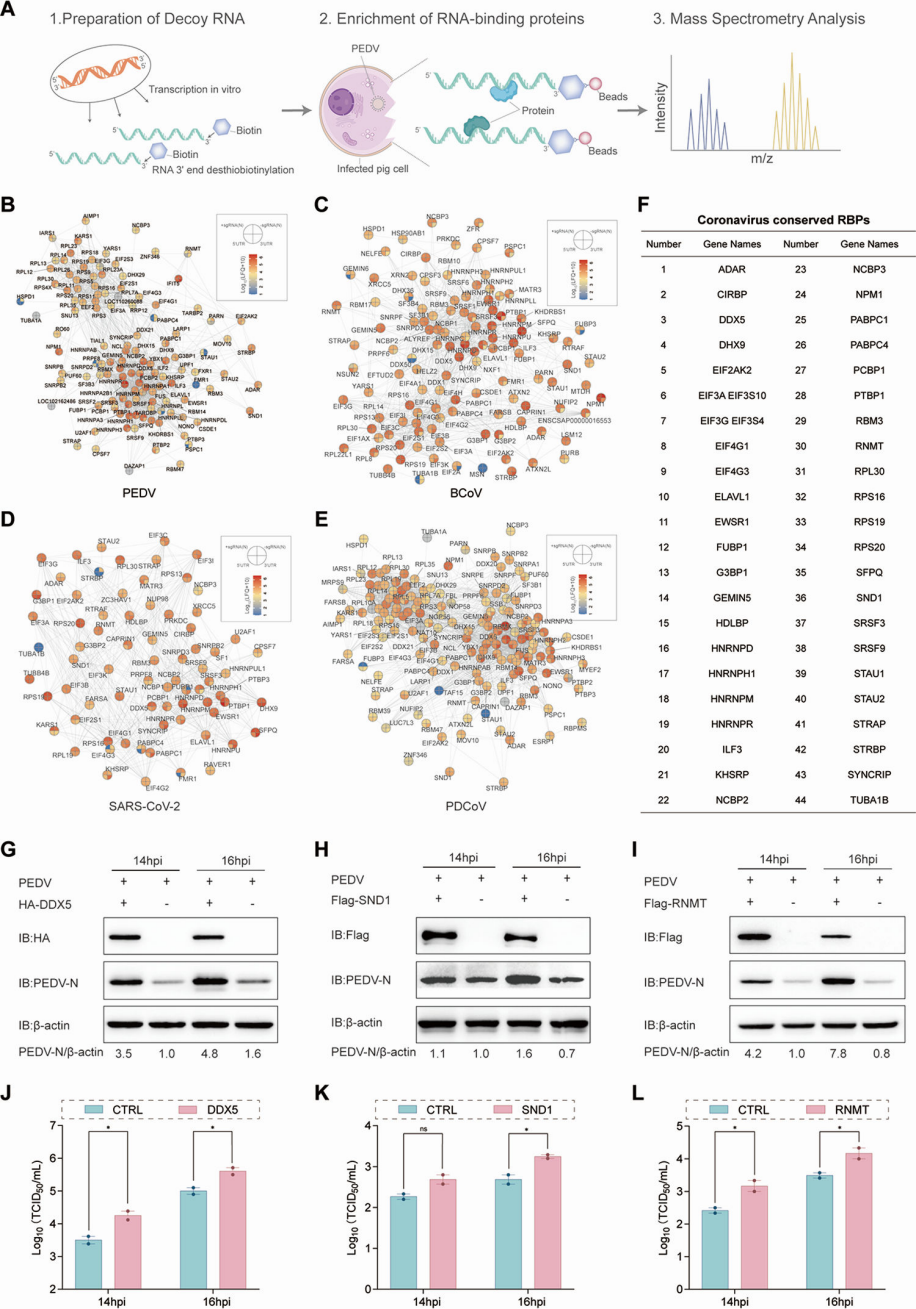
The coronavirus RNA-protein interactome at subgenome resolution. (**A**) Schematic overview of the RNA pull-down-MS workflow for identifying proteins that bind to coronavirus RNAs. (**B–E**) Coronavirus (PEDV, BCoV, SARS-CoV-2, and PDCoV) RNAs (±sgRNAs of the N, 5′-UTR, and 3′-UTR), combined with biotin, were incubated with host cell lysates for RNA pull-down and MS. Protein-protein association networks of consensus coronavirus RNA interactomes were constructed using UniProt. Pie charts and colors of nodes indicate protein enrichment across the four bait RNAs. (**F**) In the PPI networks, the intersection of 44 proteins from PEDV, BCoV, SARS-CoV-2, and PDCoV is listed by the first letter. (**G–I**) Western blotting analysis of PEDV replication in LLC-PK1 cells transfected with HA-DDX5, Flag-SND1, or Flag-RNMT plasmids. The cells were infected with PEDV (MOI = 1) and harvested at the indicated time points after viral infection. LLC-PK1 cells were transfected with HA or Flag plasmids as controls. (**J–L**) TCID_50_ analyses of PEDV replication in LLC-PK1 cells transfected with HA-DDX5, Flag-SND1, or Flag-RNMT plasmids. The cells were infected with PEDV (MOI = 1) and harvested at the indicated time points after viral infection. LLC-PK1 cells were transfected with HA or Flag plasmids as a control (CTRL). *P* values were determined by two-way ANOVA. Data are presented as mean ± SD from two replicate samples. ***P < 0.001, **P < 0.01, *P < 0.05, ns = no statistical significance.

To detect the proteinome interacting with the positive-sense subgenomes (+sgRNAs) and negative-sense subgenomes (−sgRNAs) of the N gene and the 5′-untranslated regions (5′-UTRs) and 3′-UTRs of CoVs involved in viral RNA synthesis and viral mRNA translation to facilitate viral replication, RNA pull-down and mass spectrometry (MS) were performed to screen the interaction of host proteins with ±sgRNAs of the N gene and the 5′-UTRs and 3′-UTRs of three genera: *Alphacoronavirus* (PEDV), *Betacoronavirus* (BCoV, SARS-CoV-2), and *Deltacoronavirus* (PDCoV). *In vitro* transcription was performed to obtain four kinds of RNA, including ±sgRNAs of the N gene and the 5′-UTRs and 3′-UTRs of about 100 nt fragments of the four CoVs, combined with biotin labeling, and the RNAs were incubated with lysates from host cells for RNA pull-down and MS (Fig. 1A). The MS results revealed that 1,494 host proteins interact with ±sgRNA of the N gene and the 5′-UTRs and 3′-UTRs from PEDV (784 proteins), BCoV (1056 proteins), SARS-CoV-2 (614 proteins), and PDCoV (833 proteins) (Table S1). Gene Ontology (GO) enrichment analysis identified 399 of the 1,494 proteins belonging to the RBPs (Table S2). Venn analysis revealed the intersection of 125 proteins from PEDV, 116 proteins from BCoV, 77 proteins from SARS-CoV-2, and 157 proteins from PDCoV in the interactome of ±sgRNA of the N gene and the 5′-UTRs and 3′-UTRs (Fig. S1A; Table S2). Based on the intersection of RBPs, protein-protein interaction (PPI) networks constructed using the UniProt database revealed the different abundances of proteins with different colors in the node pie charts, which were divided into four parts representing the proteins from ±sgRNA of the N gene and the 5′-UTRs and 3′-UTRs (Fig. 1B through E). In the PPI networks, the intersection of 44 proteins from PEDV, BCoV, SARS-CoV-2, and PDCoV, including DDX5, SND1, and RNMT, is listed by the first letter (Fig. 1F). Previous studies reported that DDX5 promoted the replication of SARS-CoV by interacting with nsp13 (22), PRRSV by interacting with nsp9 (23), and the influenza virus by colocalizing with viral NPs (24). They also reported that SND1 binding SARS-CoV-2 negative-sense RNA enhances viral RNA synthesis through nsp9 (3).

To analyze the roles of DDX5, SND1, and RNMT in PEDV replication, HA-tagged DDX5 (HA-DDX5), Flag-tagged SND1 (Flag-SND1), and Flag-tagged RNMT (Flag-RNMT) plasmids were transfected into LLC-PK1 cells. Following PEDV infection, samples were collected 14 h/16 h post-infection (hpi). Western blotting, RT-qPCR, and TCID_50_ titer assays revealed that the overexpression of DDX5/SND1/RNMT significantly increased viral N protein expression (Fig. 1G through I), viral N protein mRNA levels (Fig. S1B through D), and supernatant virion titers (Fig. 1J through L). Gradient concentration experiments confirmed that DDX5 and RNMT enhanced viral replication in a dose-dependent manner (Fig. S1E through H). To validate these findings, siRNA-mediated knockdown of DDX5 (siDDX5) and SND1 (siSND1) was performed. Under identical infection conditions, silencing DDX5/SND1 considerably decreased viral N protein expression (Fig. S1I and L), viral N protein mRNA synthesis (Fig. S1J and M), and virion production (Fig. S1K and N). The findings revealed that DDX5, SND1, and RNMT positively regulate PEDV replication.

### DDX5 binds a positive-sense sequence of PEDV RNA

**Fig 2:**
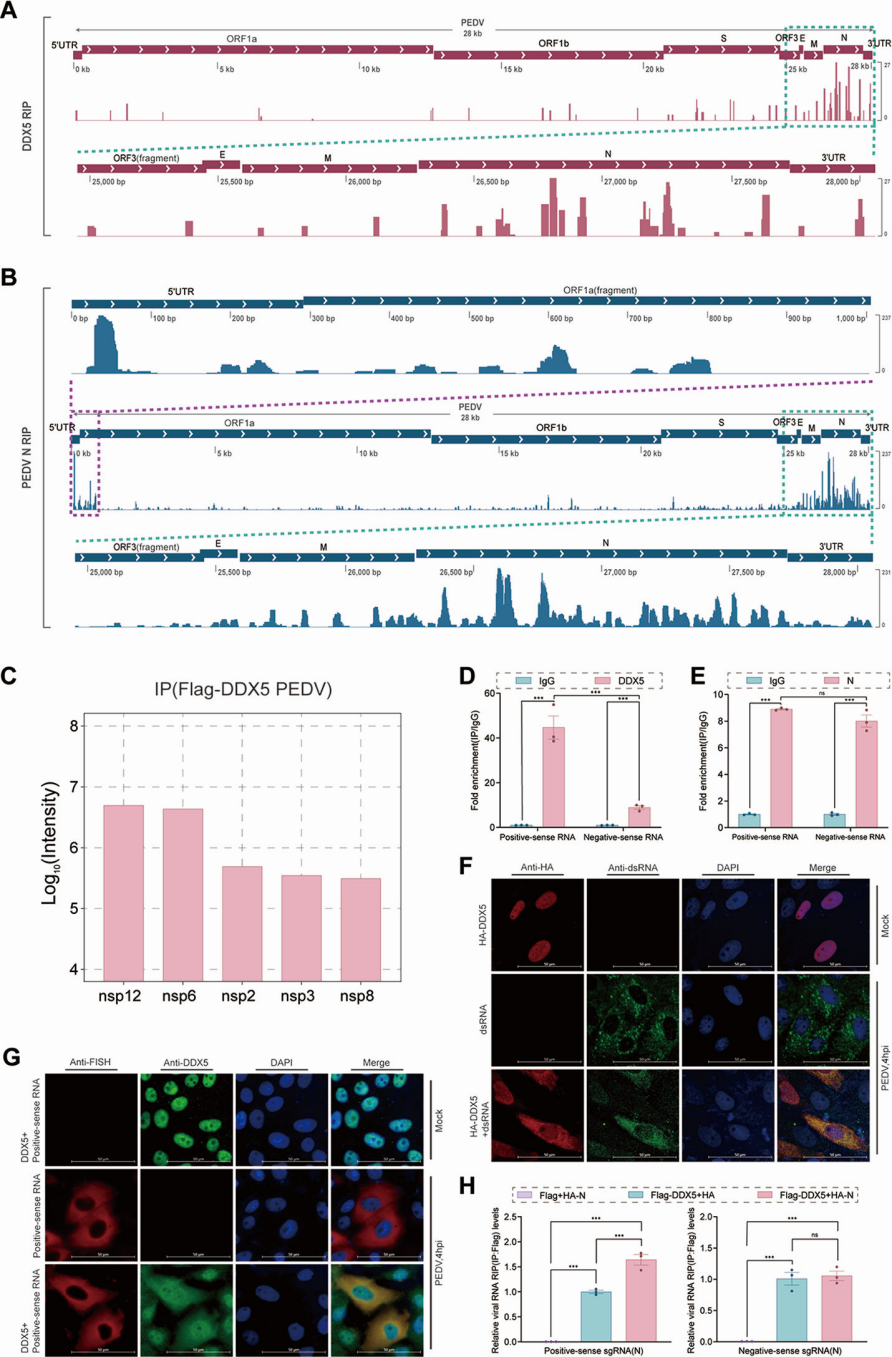
DDX5 and the viral N protein bind positive-sense PEDV RNA. (**A**) RIP-seq analysis of LLC-PK1 cells transfected with the Flag-DDX5 plasmid and infected with PEDV (MOI = 1) was performed using an anti-Flag antibody. The alignment of the RIP data with the PEDV RNA genome is shown. An enlarged view of the 3′ terminal region of the viral RNA genome is presented, with the area of interest highlighted by the green dashed boxes. (**B**) RIP-seq analysis using an anti-PEDV N antibody was conducted on PEDV-infected LLC-PK1 cells (MOI = 1). The alignment of the RIP data with the PEDV RNA genome is shown. Enlarged views of the 5′ terminal (upper) and 3′ terminal (lower) regions of the viral RNA genome are illustrated, with areas of interest marked by purple dashed boxes (upper) and green dashed boxes (lower), respectively. (**C**) A Co-IP MS assay was performed using an anti-Flag antibody in LLC-PK1 cells transfected with Flag-DDX5 or Flag plasmid (as in the CTRL) and infected with PEDV (MOI = 1). The data represent log_10_ (Intensity) fold change in the number of PEDV proteins bound to DDX5. The results were quantified relative to the CTRL (intensity >10^5^, fold change (FC) >2). (**D and E**) RIP analysis of PEDV RNA in Flag-DDX5- or Flag-N plasmid-transfected LLC-PK1 cells infected with PEDV (4 Hpi) via an anti-Flag antibody or IgG. The data are presented as fold enrichment values, with the IP-IgG group used as a negative control. Differential analysis was performed between the IP-Flag groups for positive-sense and negative-sense RNA. *P*-values were determined by one-way ANOVA. Data are presented as mean ± SD from three replicate samples. (**F**) Immunofluorescence staining of HA-DDX5 and dsRNA in PEDV-infected and HA-DDX5 plasmid-transfected LLC-PK1 cells at 4 Hpi was observed by confocal microscopy. Scale bar: 50 µm. (**G**) Immunofluorescence staining of DDX5-positive and PEDV-positive sense RNA in PEDV-infected LLC-PK1 cells at 4 Hpi was observed by confocal microscopy. Scale bar: 50 µm. (**H**) Following the transfection of HEK 293T cells with plasmids encoding Flag-DDX5, Flag, HA-N, or HA, combinations of Flag + HA-N, Flag-DDX5 + HA, and Flag-DDX5 + HA-N (1:1 ratio) were subjected to RIP analysis with *in vitro*-transcribed positive-sense sgRNA (N) or negative-sense sgRNA (N). Quantitative analysis was performed using Flag + HA-N as a blank control relative to Flag-DDX5 + HA. *P*-values were determined by one-way ANOVA. Data are presented as mean ± SD from three replicate samples. ***P < 0.001, **P < 0.01, *P < 0.05, ns = no statistical significance.

To map the DDX5 and N protein binding sites on viral RNA, we performed genome-wide native RIP-seq in LLC-PK1 cells transfected with Flag-DDX5 and Flag-N and then infected with PEDV. Sequencing analysis revealed that DDX5 was enriched specifically at the 3′ terminus of the viral genome, with binding hotspots spanning the N gene coding region and extending into the 3′-UTR (Fig. 2A, green box). The N protein bound mainly to the dual-terminal region, including the 5′-UTR, with a specific peak at nucleotides 20–60 within the 5′ leader sequence (Fig. 2B, purple box), the 3′-terminus, with peaks at the M and N genes, and the 3′-UTR (Fig. 2B, green box). The results revealed that the DDX5 and N proteins bound PEDV RNA, suggesting that DDX5 interacts with the N protein bound to the PEDV genome to promote viral replication by regulating viral RNA synthesis.

To analyze the role of DDX5 in the RTC complex of PEDV in viral RNA synthesis, coimmunoprecipitation coupled with mass spectrometry (Co-IP-MS) was performed, and the results revealed that DDX5 interacted with PEDV nonstructural proteins (nsp12, nsp6, nsp2, nsp3, and nsp8) in virus-infected cells (Fig. 2C; Table S3). Strong nsp12 and nsp6 enrichment by DDX5 in Co-IP-MS suggested that DDX5 might localize to nsp3/nsp6-mediated DMVs by binding to RTC core components to facilitate viral RNA synthesis (36, 37). Co-IP, GST pull-down, and laser scanning confocal microscopy (LSCM) revealed that DDX5 also interacted with N/nsp12/nsp9/SND1 (Fig. S2A through H and Q), SND1 with N/nsp9 (Fig. S2I through L and Q), and N interacted with nsp12/nsp9 (Fig. S2M through P, Q). The results revealed that the interactions among DDX5, SND1, N, nsp12, and nsp9 might be involved in the synthesis of PEDV RNA.

To determine the strand-binding preferences of DDX5, SND1, and N in PEDV RNA to facilitate viral RNA synthesis, RIP was used to characterize the binding features of DDX5, SND1, and N to PEDV RNA strands. In Flag-DDX5 cells infected with PEDV during the late phase of infection (20 hpi), the RIP-qPCR results revealed that DDX5 bound both PEDV positive-sense RNA and negative-sense RNA with similar efficiency and no significant difference (Fig. S3A). DDX5, N, and SND1 bound viral RNA at the early phase of infection (4 hpi), indicating that DDX5 exhibited a marked tendency to bind positive-sense viral RNA (+vRNA) (Fig. 2D) and N-bound ±vRNAs (Fig. 2E), whereas SND1 showed a preference for binding negative-sense viral RNA (−vRNA) (Fig. S3B). The results indicated that DDX5 and SND1 preferentially bind +vRNA and −vRNA, respectively, and that N binds ±vRNAs during the early phase of PEDV infection.

To elucidate the subcellular localization characteristics of DDX5 during the early phase of viral replication, colocalization analysis of HA-DDX5 and PEDV double-stranded RNA (dsRNA) using LSCM revealed significant nucleocytoplasmic translocation of DDX5 colocalized with PEDV dsRNA at 4 hpi (Fig. 2F). To validate the localization dynamics of endogenous DDX5, colocalization analysis was performed using an antibody directed against endogenous DDX5 in combination with the dsRNA. The results revealed that the nuclear export of endogenous DDX5 occurred in PEDV-infected cells and that it colocalized with dsRNA in the cytoplasm (Fig. S3C). To investigate the direct interaction between DDX5 and viral RNA, RNAscope multiplex fluorescence *in situ* hybridization was performed to detect the subcellular location of DDX5 and PEDV positive-sense RNA, and significant colocalization between endogenous DDX5 and positive-sense RNA of the N gene in the cytoplasm was detected at 4 hpi (Fig. 2G). These findings suggest that PEDV infection induces the nucleocytoplasmic translocation of DDX5, which binds PEDV positive-sense RNA to facilitate the synthesis of viral RNA.

To detect the enhanced binding of viral RNA by the interaction of DDX5, SND1, and N, a specific target protein-focused *in vitro*-transcribed RNA immunoprecipitation (STPIVT-RIP) was designed (Fig. S3D) and used to test whether the interaction of the DDX5, SND1, and N proteins facilitated the binding of PEDV RNA. The results revealed that the N protein promoted DDX5 binding to the +sgRNA of PEDV, with no effect on −sgRNA (Fig. 2H), and advanced SND1 binding to the −sgRNA of PEDV, with no effect on +sgRNA (Fig. S3E). SND1 enhanced the binding of DDX5 to the ±sgRNA of PEDV (Fig. S3F), suggesting that the interaction of DDX5, SND1, and the N protein coregulated the synthesis of viral RNA during replication.

### DDX5 promotes PEDV RNA synthesis

**Fig 3:**
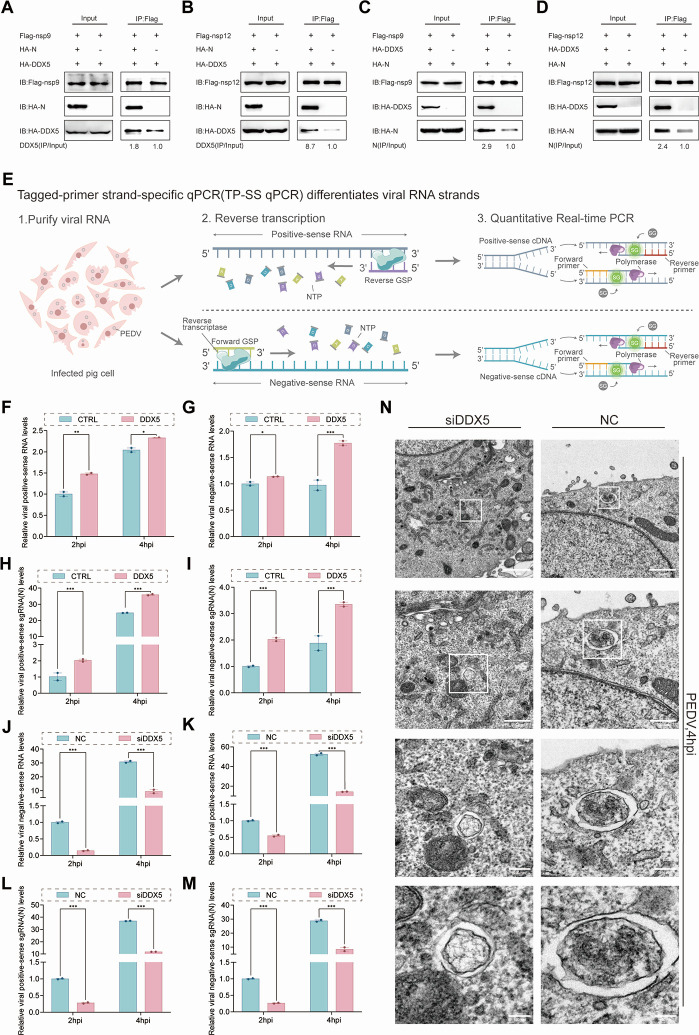
DDX5 promotes PEDV RNA synthesis. (**A and B**) Co-IP analysis of the effects of N on the interactions between nsp9 and DDX5 or between nsp12 and DDX5. Lysates from HEK 293T cells transfected with Flag-nsp9, Flag-nsp12, HA-DDX5, HA-N, or HA plasmids were mixed at a 1:1:1 ratio. (**C and D**) Co-IP analysis of the effect of DDX5 on the interaction between nsp9 and N, nsp12, and N. Lysates from HEK 293T cells transfected with Flag-nsp9, Flag-nsp12, HA-DDX5, HA-N, or HA plasmids were mixed at a 1:1:1 ratio. (**E**) Schematic illustration of the tagged-primer strand-specific qPCR (TP-SS qPCR) workflow enabling specific discrimination between viral positive-sense and negative-sense RNA levels. (**F–I**) TP-SS qPCR analysis of PEDV positive-sense RNA, negative-sense RNA, positive-sense sgRNA (N) and negative-sense sgRNA (N) levels in HA- or HA-DDX5-transfected LLC-PK1-infected cells affected by PEDV (MOI = 200). The data were normalized to GAPDH as an internal reference and quantified relative to the HA group at 2 Hpi. *P*-values determined by two-way ANOVA. Data are presented as mean ± SD from two replicate samples. (**J–M**) TP-SS qPCR analysis of PEDV positive-sense RNA, negative-sense RNA, positive-sense sgRNA (N), or negative-sense sgRNA (N) levels in LLC-PK1 cells transfected with siDDX5 or NC and infected with PEDV (MOI = 200). The data were normalized to GAPDH as an internal reference and quantified relative to the NC group at 2 Hpi. *P*-values determined by two-way ANOVA. Data are presented as mean ± SD from two replicate samples. (**N**) Computational images of representative electron tomograms of DDX5-knockdown (siDDX5) and control (NC) cells infected with PEDV (MOI = 200). A zoomed-in image of the region containing DMVs is shown at different magnifications. Upper: scale bars, 1 μm; middle: scale bars, 500 nm and 200 nm; lower: scale bars, 100 nm. ***P < 0.001, **P < 0.01, *P < 0.05, ns = no significance.

An enhanced interaction coimmunoprecipitation (EI-CoIP) assay (Fig. S4A) was performed to assess the effects of the presence or absence of N/DDX5 on the interaction efficiency with SND1/nsp9/nsp12. The results revealed that DDX5-nsp9/nsp12/SND1 and SND1-nsp9 binding significantly increased in N-containing lysates (Fig. 3A and B; Fig. S4B and C), and N-nsp9/nsp12/SND1 and SND1-nsp9 interactions were enhanced by DDX5 (Fig. 3C and D; Fig. S4D and E). These data suggest that N and DDX5 act as molecular hubs bridging SND1/nsp12/nsp9 and that DDX5/SND1 coordinates with N/nsp12/nsp9 to assemble RTCs to assist in the synthesis of PEDV RNA.

**Fig 4:**
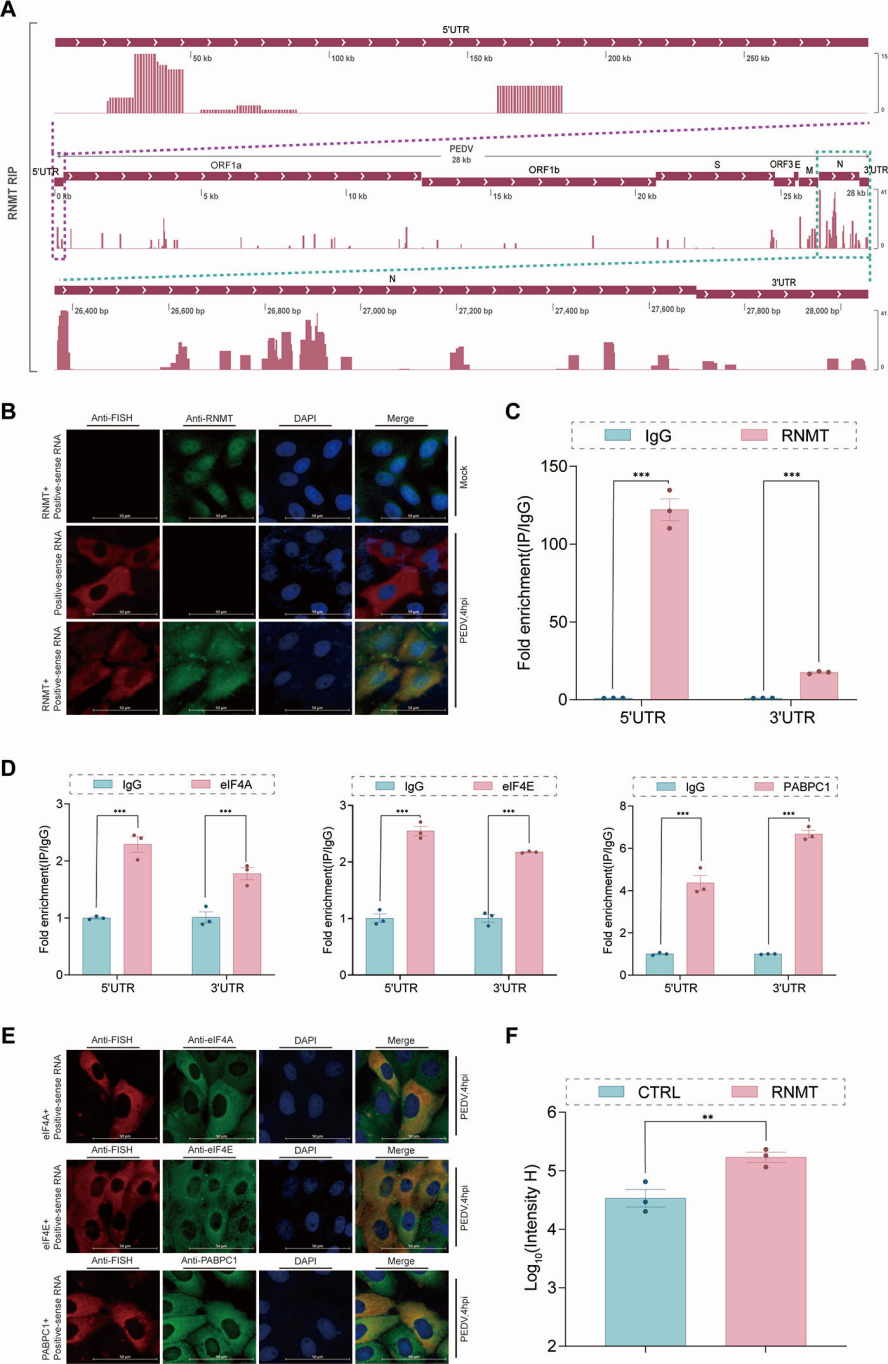
RNMT facilitates PEDV protein translation. (**A**) RIP-seq analysis of LLC-PK1 cells transfected with the Flag-RNMT plasmid and infected with PEDV (MOI = 1) was performed using an anti-Flag antibody. The alignment of the RIP data with the PEDV RNA genome is shown. Enlarged views of the 5′ terminal (upper) and 3′ terminal (lower) regions of the viral RNA genome are illustrated, with areas of interest marked by purple dashed boxes (upper) and green dashed boxes (lower), respectively. (**B**) Immunofluorescence staining of RNMT- and PEDV-positive sense RNA in PEDV-infected LLC-PK1 cells at 4 hpi was observed via confocal microscopy. Scale bar: 50 µm. (**C**) RIP analysis of PEDV RNA using an anti-Flag antibody or IgG in Flag-RNMT plasmid-transfected LLC-PK1 cells infected with PEDV (4 hpi). Differential analysis was performed for RNMT binding to the 5′ UTR and 3′ UTR of positive-sense RNA. *P*-values were determined by one-way ANOVA. Data are presented as mean ± SD from three replicate samples. (**D**) RIP analysis of PEDV RNA in Flag- eIF4A/eIF4E/PABPC1 plasmid-transfected LLC-PK1 cells infected with PEDV (4 hpi) using an anti-Flag antibody or IgG. Differential analysis was performed for eIF4A/eIF4E/PABPC1 binding to the 5′ UTR and 3′ UTR of positive-sense RNA. *P*-values determined by one-way ANOVA. Data are presented as mean ± SD from three replicate samples. (**E**) Immunofluorescence staining of eIF4A/eIF4E/PABPC1 and PEDV positive-sense RNA in PEDV-infected LLC-PK1 cells at 4 hpi was performed via confocal microscopy. Scale bar: 50 µm. (**F**) Co-IP-MS analysis of PEDV N protein with heavy-chain amino acids (^13^C_6_-Lys/^13^C_6_-Arg) using the PEDV N antibody in Flag-RNMT or Flag plasmid-transfected LLC-PK1 cells infected with PEDV (1 hpi). The data show Log_10_(^Intensity H^) fold change in the number of heavy-chain amino acids in the PEDV N protein. *P*-values determined by one-way ANOVA. Data are presented as mean ± SD from three replicate samples. ***P < 0.001, **P < 0.01, *P < 0.05, ns = not significant.

To confirm that DDX5 and SND1 promote PEDV replication by assisting in viral RNA synthesis, HA-DDX5- and Flag-SND1 were transfected into LLC-PK1 cells, which were infected with PEDV at an MOI of 200, 2 hpi to detect viral RNA by tagged-primer strand-specific qPCR (TP-SS qPCR) (Fig. 3E). The cells were collected after 2 h and 4 h of PEDV infection to detect +vRNA, −vRNA, +sgRNA, and −sgRNA by TP-SS qPCR. The results revealed that DDX5 increased the synthesis of ±vRNA at 2 hpi (Fig. 3F and G). Compared to DDX5, SND1 preferentially promoted −vRNA synthesis at 2 hpi (Fig. S4G), whereas it increased +vRNA synthesis at 4 hpi (Fig. S4F). The findings revealed that DDX5 and SND1 play different roles in the synthesis of PEDV RNA. To detect the production of PEDV sgRNA during discontinuous transcription, specific fluorescent quantitative primers were designed to span the junction between the sgRNA leader sequence and the gene body. We found that both ±sgRNA values were significantly increased in HA-DDX5 cells at 2 hpi (Fig. 4H and I). In Flag-SND1 cells, the effect of −sgRNA markedly increased at 2 hpi (Fig. S4I), whereas the enhancing effect of +sgRNA was delayed until 4 hpi (Fig. S4H), which confirmed the critical role of SND1 in the synthesis of sgRNA. At 2 and 4 hpi, DDX5 knockdown significantly reduced both ±vRNA and both ±sgRNA (Fig. 3J through M). These findings showed that DDX5 and SND1 are crucial host factors that assist in viral RNA synthesis for PEDV replication.

The double-membrane vesicles (DMVs) induced by CoVs are platforms for viral replication (38), and the DMVs induced by SARS-CoV-2 are smaller in SND1 knockdown cells (39). To detect whether DDX5 is involved in DMV, transmission electron microscopy (TEM) was performed to observe DMVs in PEDV-infected DDX5-knockdown cells at 4 hpi. Compared to negative control (NC) cells, DDX5 knockdown cells presented DMVs with significantly smaller cross-sectional areas (Fig. 3N). A decrease in the size of DMVs in DDX5 knockdown cells may be associated with reduced production of viral RNA, regulated by a decrease in DDX5 levels.

### RNMT facilitates PEDV protein translation

The enzyme RNMT, a guanine-N7 MTase, binds viral RNA to promote PEDV replication. We found that RNMT interacted with N, DDX5, SND1, nsp12, and nsp14 (Fig. S5A through J and M) and that N interacted with nsp14 (Fig. S5K through L, M) via Co-IP, GST pull-down, and LSCM, which are RBPs that are important components of the RTC that assist in viral RNA synthesis. The results revealed that RNMT and N were located within the RTCs of PEDV, suggesting that the interaction of RNMT and nsp14 with RTCs capped viral RNA immediately after synthesis to improve capping efficiency.

To map RNMT binding sites on viral RNA, we performed genome-wide native RIP-seq in LLC-PK1 cells transfected with Flag-RNMT and then infected with PEDV. The results of sequencing analysis revealed that RNMT bound to the 5′ terminus of the viral genome, with a specific peak at nucleotides 20–50 within the 5′ leader sequence, and to the 3′ terminus of the viral genome, with peaks at N genes and 3′-UTRs (Fig. 4A). To elucidate the subcellular localization characteristics of RNMT with the dsRNA of PEDV, colocalization analysis of RNMT and PEDV dsRNA using LSCM revealed that RNMT colocalized with the dsRNA of PEDV at 4 hpi (Fig. S6A). To investigate the direct interaction of RNMT with viral RNA, RNAscope multiplex fluorescence *in situ* hybridization revealed that RNMT significantly localized with positive-sense RNA of PEDV in the cytoplasm at 4 hpi (Fig. 4B). These findings suggested that RNMT may bind the dual terminus of PEDV positive-sense RNA. To confirm that RNMT binds the dual termini of PEDV positive-sense RNA, RIP-qPCR assays revealed that RNMT and nsp14 exhibited a strong tendency to bind PEDV positive-sense RNA (Fig. S6B and C), which further revealed that RNMT and nsp14 bound both the 5′-UTR and 3′-UTR (Fig. 4C; Fig. S6D). An EI-CoIP assay revealed that RNMT-nsp14 binding significantly increased in N-containing lysates (Fig. S6G). RNMT and nsp14 are known as guanine-N7 MTases that cap RNA and interact with N and both the 5′-UTR and 3′-UTR, which might cyclize mRNAs to facilitate protein translation.

The N protein of PEDV captures eIF4A, eIF4E, and PABPC1 to cyclize viral mRNA, assisting in viral transcription to promote viral replication (40). The interactions of RNMT with eIF4A, eIF4E, and PABPC1 were confirmed by Co-IP, GST pull-down, and LSCM (Fig. S7A through G). RIP-qPCR assays revealed that eIF4A, eIF4E, and PABPC1 tended to bind PEDV positive-sense RNA (Fig. S6F), and eIF4A, eIF4E, and PABPC1 bound both the 5′-UTR and 3′-UTR (Fig. 4D). The subcellular localization of eIF4A, eIF4E, and PABPC1 with dsRNA of PEDV revealed that they colocalized with dsRNA of PEDV at 4 hpi (Fig. S6E), and RNAScope multiplex fluorescence *in situ* hybridization revealed that eIF4A, eIF4E, and PABPC1 localized with positive-sense RNA of PEDV in the cytoplasm at 4 hpi (Fig. 4E). EI-CoIP assays revealed that RNMT promoted the interaction of PABPC1 with eIF4A and eIF4E (Fig. S6H and I). The results showed that N and RNMT carrying viral mRNA might capture eIF4A, eIF4E, and PABPC1 to cyclize viral mRNA using the host protein translation system to efficiently promote viral protein translation for PEDV replication. To confirm that RNMT enhances viral protein translation, LLC-PK1 cells were cultured with serum without lysine and arginine supplemented with ^13^C6-L-arginine and ^13^C6-L-lysine, which were subsequently transfected with Flag-RNMT or Flag and infected with PEDV, and the N protein of PEDV in the cell lysate was enriched by CoIP for detection via MS. The results revealed that RNMT significantly promoted the labeling of proteins with ^13^C6-L-arginine and ^13^C6-L-lysine (Fig. 4F), suggesting that RNMT participated in protein translation to facilitate PEDV replication.

### DDX5 promotes CoV replication

**Fig 5:**
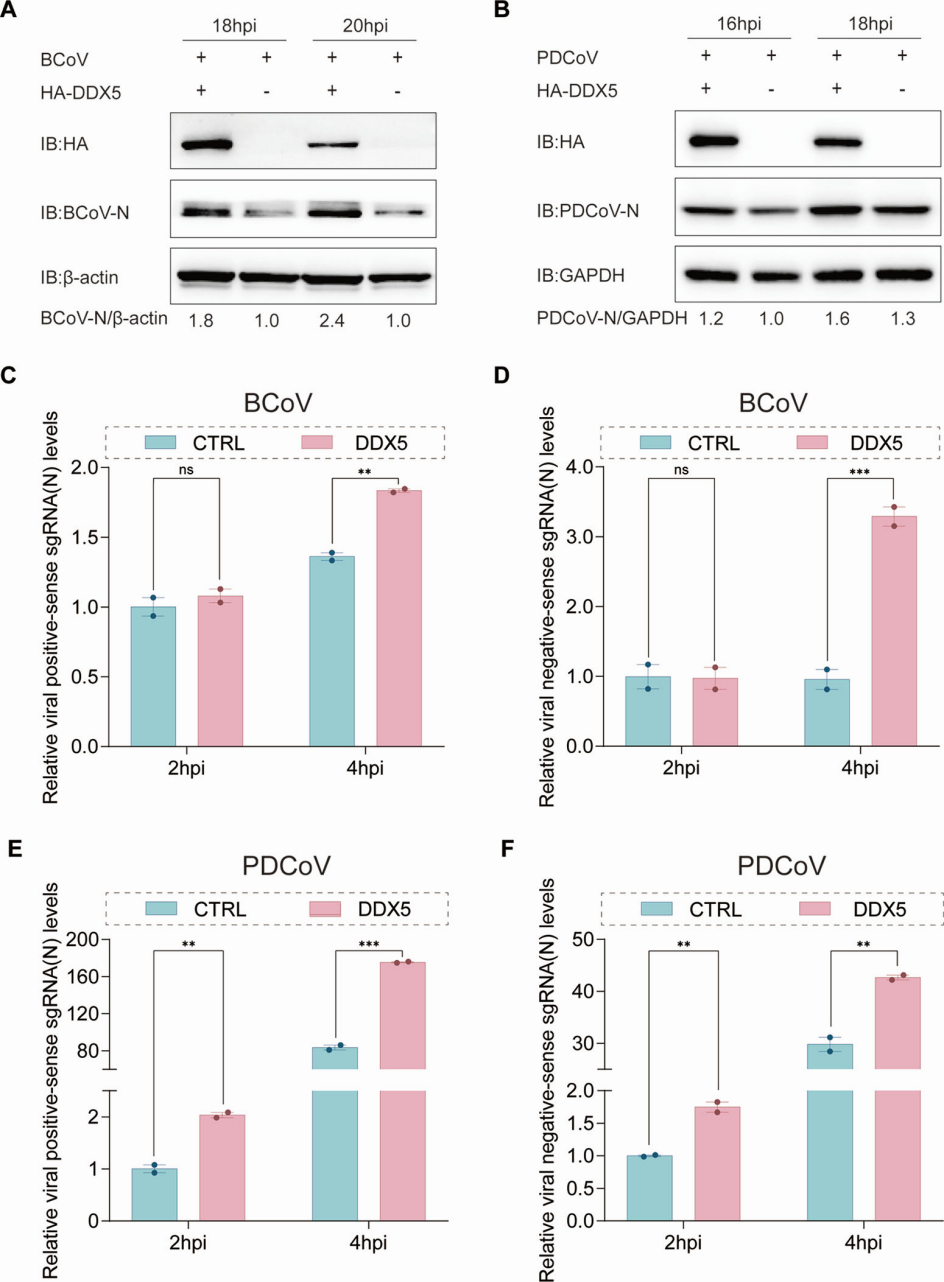
DDX5 promotes coronavirus replication. (**A**) Western blotting analysis of BCoV N protein expression in Vero cells transfected with HA or HA-DDX5 and infected with BCoV (MOI = 1). b-actin served as a loading control. (**B**) Western blotting analysis of PDCoV N protein expression levels in LLC-PK1 cells transfected with HA or HA-DDX5 and infected with PDCoV (MOI = 1). GAPDH served as a loading control. (**C and D**) TP-SS qPCR analysis of BCoV-positive sgRNA (N) and negative-sense sgRNA (N) levels in HA- or HA-DDX5-transfected Vero cells infected with BCoV (MOI = 200). GAPDH served as an internal control, with quantification relative to the HA group (CTRL) after infection for 2 h. *P*-values determined by two-way ANOVA. Data are presented as mean ± SD from two replicate samples. (**E and F**) TP-SS qPCR analysis of PDCoV-positive sgRNA (N) and negative-sense sgRNA (N) levels in HA- or HA-DDX5-transfected LLC-PK1 cells infected with PDCoV (MOI = 200). GAPDH served as an internal control, with quantification relative to the HA group (CTRL) after infection for 2 h. *P*-values determined by two-way ANOVA. Data are presented as mean ± SD from two replicate samples. ***P < 0.001, **P < 0.01, *P < 0.05, ns = not significant.

The capture of DDX5, SND1, and RNMT by the N protein facilitates PEDV RNA synthesis and protein translation, and whether DDX5, SND1, and RNMT interact with the N proteins of *Betacoronavirus* (BCoV, SARS-CoV, SARS-CoV-2, and MERS-CoV), *Gammacoronavirus* (IBV), and *Deltacoronavirus* (PDCoV) to assist in viral replication was determined. The results of Co-IP assays revealed that DDX5 interacted with the N proteins of BCoV, PDCoV, SARS-CoV, SARS-CoV-2, MERS-CoV, and IBV (Fig. S8A through F). To confirm whether DDX5 broadly assisted the replication of CoVs, HA-DDX5 plasmids were translated into Vero and LLC-PK1 cells infected with BCoV and PDCoV, respectively. The samples collected at 18/20 hpi with Vero cells infected with BCoV and 16/18 hpi with LLC-PK1 cells infected with PDCoV were detected by western blotting and RT-qPCR assays. The results showed that DDX5 significantly promoted the replication of BCoV and PDCoV (Fig. 5A and B; Fig. S8G and H). These results suggested that DDX5 might participate in viral RNA synthesis in the three genera of CoVs. To reveal that DDX5 promotes BCoV and PDCoV replication by assisting in viral RNA synthesis, TP-SS qPCR assays were performed. The results revealed that DDX5 significantly promoted ±vRNA and ±sgRNA of BCoV at 4 hpi (Fig. 5C and D; Fig. S8I and J), as well as ±vRNA and ±vsgRNA of PDCoV at 2 hpi (Fig. 5E and F; Fig. S8K and L), suggesting that DDX5 broadly assisted in viral RNA synthesis in CoVs for replication.

## DISCUSSION

**Fig 6:**
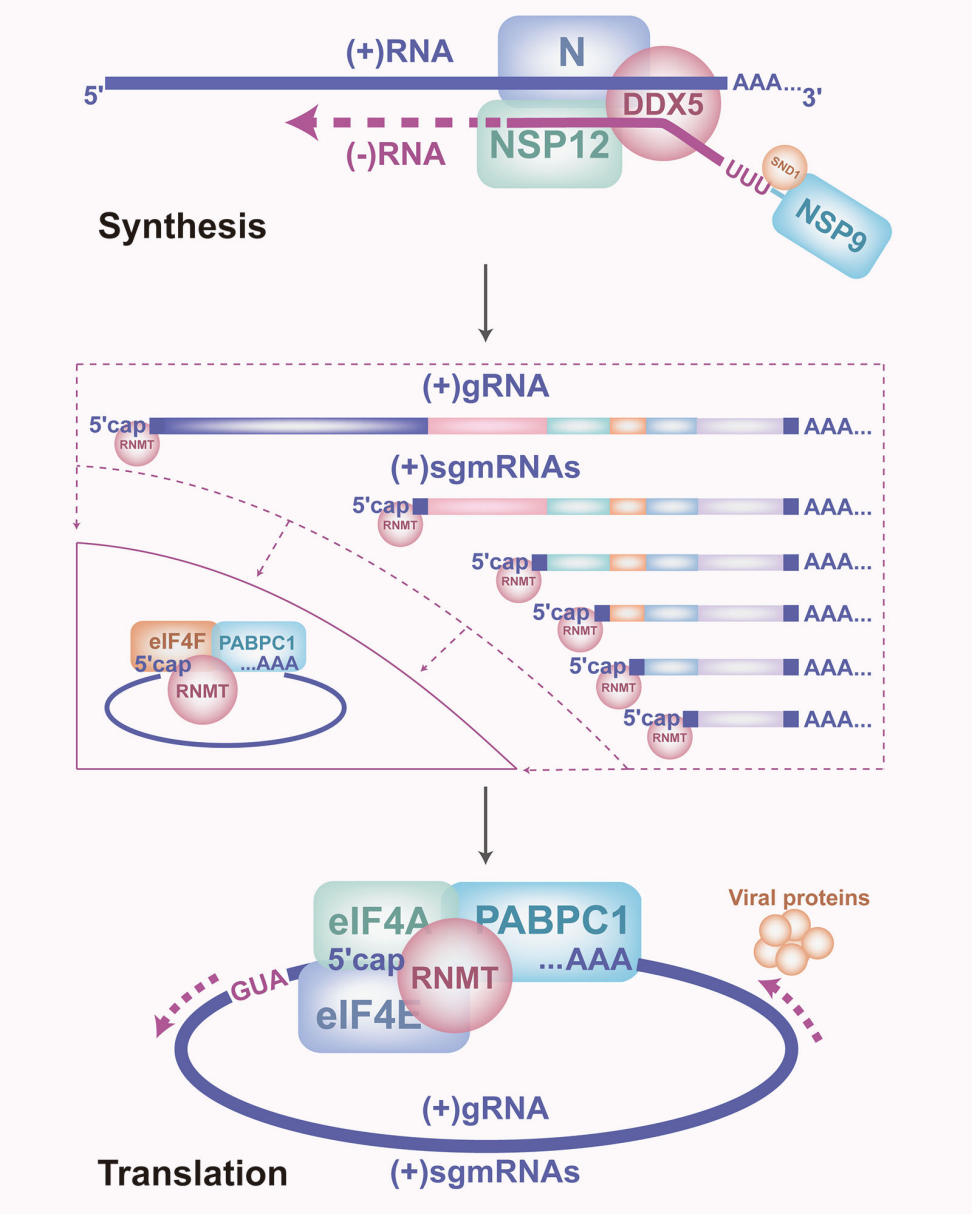
DDX5 and RNMT facilitate viral RNA synthesis and viral protein translation. The N protein captures the host proteins DDX5, SND1, and RNMT, cooperates with DDX5 binding +vRNA and SND1 binding −vRNA, and interacts with nsp9 and nsp12 to drive RTC complex assembly to assist in viral RNA synthesis. Subsequently, RNMT hijacks a host protein translation system to cyclize viral mRNA to assist in viral protein translation.

Emerging and recurring CoVs have threatened humans and livestock in the last few years. CoVs use viral proteins and host proteins to synthesize viral RNA and proteins (41, 42). Analyzing the synthesis of CoV RNA and proteins can help develop therapeutic drugs (9). In this study, we revealed that the N protein captures the host proteins DDX5, SND1, and RNMT, which interact with the sgRNA of the N gene and the 5′-UTRs and 3′-UTRs of CoVs to promote virus replication. DDX5 by binding +vRNA and SND1 by binding −vRNA, and interacts with nsp9 and nsp12 to drive RTC complex assembly to assist in viral RNA synthesis. Subsequently, RNMT hijacks a host protein translation system to cyclize viral mRNA to assist in viral protein translation (Fig. 6).

In CoV, RNA synthesis is an important step in the viral life cycle (43). RTCs are mainly responsible for the synthesis of viral RNA. The core CoV proteins of RTCs synthesize viral RNA (5), but the host factors involved in RTC assembly to assist in RNA synthesis are unclear; however, some studies have shown that DDX1 facilitates the genome transition of mouse hepatitis virus (MHV) from discontinuous to continuous transcription (6), and SND1 binds the 5′ end of the vRNA of SARS-CoV-2 RNA to assist in viral RNA synthesis through interactions with nsp9, which affects the DMV size induced by SARS-CoV-2(3). This study revealed that the proteinome interacts with the ±sgRNA of the N gene and the 5′-UTRs and 3′-UTRs of PEDV, BCoV, SARS-CoV-2, and PDCoV. It also found that 44 host RBPs, including DDX5, SND1, and RNMT, broadly interact with coronaviral RNA. DDX5, SND1, and RNMT positively regulate PEDV replication, and DDX5 affects the size of DMVs, which might be related to the production of viral RNA regulated by DDX5. DDX5 is a member of the DEAD box family of RNA helicases that participate in transcription regulation and interact with viral proteins to promote the replication of SARS-CoV, HIV-1, HCV, JEV, PRRSV, and influenza virus (22, 23, 44–48). SND1, initially known as a transcriptional coactivator that interacts with Epstein-Barr virus nuclear antigen 2, binds the negative-sense RNA of SARS-CoV-2 to increase viral RNA synthesis (3, 49, 50). DDX5 and SND1 may assist PEDV RNA synthesis to regulate the replication of PEDV. RNMT, as a guanine-N7 MTase and RBP, participates in the last step of capping mRNA, but the mechanism by which RNMT participates in viral proliferation is unclear.

The core coronaviral proteins of RTCs that synthesize viral RNA and add caps to the 5′ end of viral RNA include nsp12/9/8/7/13/14/16 (5, 31, 36, 51–55). This study revealed that DDX5 shuttles from the nucleus to the cytoplasm and interacts with PEDV nonstructural proteins (nsp12/6/2/3/8/9). SND1 shuttling between the nucleus and cytoplasm interacts with N/nsp9, and N interacts with nsp12/9, suggesting that DDX5 and SND1 are components of RTCs that assist in PEDV RNA synthesis to regulate PEDV replication. The results of the EI-CoIP assay revealed that the DDX5 and N proteins enhanced the interaction between the components of RTCs to regulate PEDV replication, suggesting that DDX5 and N are molecular hubs bridging SND1/nsp12/nsp9 and that DDX5/SND1 coordinates with the viral N and nsp12/9 proteins to assemble RTCs to assist in the synthesis of PEDV RNA.

The binding of RTC to viral RNA is the foundation of viral RNA synthesis (56). SND1 interacts with nsp9 and binds to the gRNA of SARS-CoV-2 to promote viral RNA synthesis, thereby influencing the size of DMVs (3). DDX5 and N are components of PEDV RTCs. The results revealed that DDX5 bound mainly to the genes encoding structural proteins and the 3′-UTR of the PEDV genome, the N protein bound to the 5′-UTR with a specific peak, and the genes encoding structural proteins had more peaks at the N genes. Additionally, DDX5 bound to the ±vRNA of PEDV with similar efficiency during the late phase of infection (20 hpi), but DDX5 bound mainly to +vRNA, SND1 bound mainly to -vRNA, and the N protein bound ±vRNAs at the early phase of infection (4 hpi), suggesting that the N protein-capturing host proteins (DDX5 and SND1) bind ±vRNA to assist the synthesis of PEDV RNA.

The N protein captures DDX5 binding +vRNA and SND1 binding −vRNA to interact with nsp9 and nsp12, which are the core CoV proteins of RTCs that participate in viral RNA synthesis. The TP-SS qPCR results revealed that DDX5 promoted the synthesis of +vgRNA and that SND1 enhanced −vgRNA synthesis at 2 hpi, suggesting that DDX5 and SND1 play different roles in viral RNA synthesis. The sgRNA results revealed that the number of both ±sgRNAs was increased by DDX5, whereas the number of −sgRNA was increased by SND1 at 2 hpi. DMVs induced by CoVs are platforms for viral replication, and the sizes induced by SARS-CoV-2 are smaller in SND1 KO cells (39). The number of DMVs induced by PEDV was lower in DDX5 knockdown cells. These results showed that DDX5 participated mainly in ±sgRNA synthesis, whereas SND1 participated in −sgRNA synthesis, suggesting that DDX5 and SND1, which are captured by the N protein and play different roles in viral replication, are crucial host factors that assist in viral RNA synthesis during PEDV replication.

As guanine-N7 MTases and RBPs, RNMT, and nsp14, respectively, participate in the last step of capping mRNA. Capped RNA is recognized by eIF4F complexes, including eIF4A, eIF4E, and eIF4G, which interact with PABP to bind to poly(A) of mRNAs, forming a loop structure to promote protein synthesis (57). The interaction of the N protein of PEDV with PABPC1 and eIF4F promotes viral replication (40). This report revealed that RNMT interacts with host proteins (DDX5 and SND1) and viral proteins (N, nsp14, nsp12, and nsp9), which are important components of the RTC that assist in viral RNA synthesis and capping of viral RNA (5, 30–32). As the guanine-N7 MTase of CoVs, nsp14 enhances the interaction between RNMT and the N of PEDV. We found that RNMT and nsp14 were located within the RTCs of PEDV, suggesting that RNMT and nsp14 interact with RTC-capped viral RNA immediately after synthesis to improve capping efficiency and that RNMT might be captured to assist in capping viral RNA. RNMT, which binds the genome of PEDV at the 5′-UTR and 3′-UTR, interacts with N and interacts with eIF4A, eIF4E, and PABPC1, suggesting that RNMT may cyclize mRNAs to efficiently facilitate protein translation after viral RNA synthesis and capping. The finding that RNMT promotes the nascent protein labeled with ^13^C6-L-arginine and ^13^C6-L-lysine confirmed that RNMT participates in protein translation to facilitate PEDV replication (58).

Host proteins regulating pancoronavirus proliferation are targets for developing broad antiviral drugs. The host protein ARF1 is a potential target for developing anti-pan-SARS-CoV-2 drugs (58), and the host protein AP1 is a candidate target for developing broad antiviral drugs (59). This study revealed that DDX5, which interacts with host proteins (SND1 and RNMT) and viral proteins (N, nsp2, nsp3, nsp6, nsp8, nsp9, and nsp12), binds viral RNA to facilitate viral RNA synthesis for PEDV replication. It broadly interacts with N proteins of CoVs, including *Betacoronavirus* (BCoV, SARS-CoV, SARS-CoV-2, and MERS-CoV), *Gammacoronavirus* (IBV), and *Deltacoronavirus* (PDCoV), to promote viral replication. DDX5 might be a target for developing antiviral drugs to prevent it from interacting with coronaviral proteins to inhibit CoV replication.

To summarize, we found that DDX5, SND1, and RNMT promote PEDV replication via the interactomes of CoV RNA-protein. DDX5 and SND1 interact with RTCs to promote PEDV RNA synthesis by DDX5 binding +vgRNA and SND1 binding −vgRNA, and RNMT hijacks the host protein translation system to cyclize the viral mRNA, thereby facilitating viral protein translation. The results revealed that the N protein can capture DDX5, facilitating viral RNA synthesis, and that RNMT promotes viral protein translation for CoV replication, which are the potential targets for antiviral tactics.

## MATERIALS AND METHODS

### Cell culture

We used HEK 293T (human) cells, Vero (African green monkey) cells, and HeLa (human) cells cultured in DMEM supplemented with 10% heat-inactivated fetal bovine serum (FBS). LLC-PK1 (porcine) cells were cultivated in MEM supplemented with 10% heat-inactivated FBS. All cells were maintained at 37 °C and a CO_2_ concentration of 5% in a constant-temperature incubator.

### Plasmids

Eukaryotic plasmids were constructed by cloning porcine DDX5 (XM_021066761.1) and porcine SND1 (XM_021078633.1) with a C-terminal Flag tag or HA tag into the pXJ41 vector using the ClonExpress II One Step Cloning Kit (Vazyme Biotech, C112-01). Next, PEDV NSP9 with a C-terminal Flag tag was cloned and inserted into the pEGFP vector. Other eukaryotic plasmids containing PEDV N and PEDV NSP12 were stored in the laboratory. Additionally, for prokaryotic expression plasmid construction, genes with a C-terminal Flag tag were cloned and inserted into the pCold-TF (Clontech Laboratories, Inc., 3365) or pCold-GST (Clontech Laboratories, Inc., 3372) vectors.

### Transfection

The cells were cultured in 12-well plates, followed by plasmid transfection using Lipofectamine 3000 until the cell confluence reached 70%–90%. Additionally, Lipofectamine RNAiMAX was used to transfect the siRNAs when the cells reached 50%–60% confluence. The primer sequences are provided in Table S4.

### Virus infections

LLC-PK1 cells were used for PEDV (60) and PDCoV infection, and Vero cells were used for BCoV infection. All cells were infected with the virus when they reached over 90% confluence. The viral inoculum was prepared with MEM or DMEM containing 3 µg/mL trypsin (0.25%) and 100 U/mL penicillin-streptomycin. After the cells were washed with PBS, the virus-containing inoculum was added, and the mixture was incubated at 37 °C for 1 h. The inoculum was then removed, the cells were washed with PBS, and the mixture was replaced with fresh virus-free inoculum for continued culture. As previously described, Vero cells were used for PEDV titration (60).

### RNA pull-down assay

To prepare templates for *in vitro* transcription, we designed forward primers containing the T7 promoter and corresponding reverse primers. The PCR products were purified and used as templates for *in vitro* transcription. *In vitro* transcription was performed using the EasyCap T7 Cotranscription Kit with a CAG Trimer (Vazyme Biotech, DD4203), followed by RNA purification with VAHTS RNA Clean Beads (Vazyme Biotech, N412). The purified RNA was 3′-end desthiobiotinylated using the Pierce RNA 3′ End Desthiobiotinylation Kit (Thermo Fisher Scientific, 20163), and the labeled RNA was stored at −80 °C.

The cells were cultured in T25 flasks until they reached 90% confluence and were subsequently lysed on ice for 30 min using Pierce IP lysis buffer (Thermo Fisher Scientific, 87787) containing a protease inhibitor cocktail, followed by scraping. The lysates were centrifuged at 4 °C and 12,000 rpm for 5 min, and the supernatants were stored at −80 °C for subsequent use. Following the manufacturer’s protocol, the Pierce Magnetic RNA-Protein Pull-Down Kit (Thermo Fisher Scientific, 20164) was used to capture RBPs using labeled RNA, with the following optimizations: 100 U of RNase extract was added and mixed uniformly during both the target RNA-magnetic bead binding and RNA-RBP interaction stages. For the eluted RBP complexes, 50 μL of SDT buffer (61) was added for mass spectrometry analysis.

Protein quantification was performed using the Bicinchoninic Acid Kit for Protein Determination (Sigma-Aldrich, BCA1) following the manufacturer’s instructions. Filter-aided proteome preparation (FASP) (61) was employed for tryptic digestion of proteins from each sample. Each sample was separated using the nanoflow HPLC system NanoElute (Bruker Daltonics, Germany). Mobile phase A was 0.1% formic acid aqueous solution, and mobile phase B was 0.1% formic acid in 84% acetonitrile aqueous solution. The column was equilibrated with 95% mobile phase A. Samples were loaded onto the loading column via an autosampler and separated on the analytical column at a flow rate of 300 nL/min (62). After chromatographic separation, the samples were analyzed using a Tims TOF Pro (4D) mass spectrometer (Bruker Daltonics, Germany). Detection was performed in positive ion mode with the ion source voltage set to 1.5 kV. Both MS and MS/MS spectra were acquired and analyzed using the TOF detector. The mass scanning range was set to 100–1,700 m/z. Data acquisition utilized the parallel accumulation-serial fragmentation (PASEF) mode. Following one full MS scan, 10 PASEF MS/MS scans were performed per cycle with a cycle window time of 1.17 s. For MS/MS scans with precursor charges ranging from 0 to 5, a dynamic exclusion time of 24 s was applied to prevent repeated scanning of the same parent ions.

### Western blotting analysis

The cells were lysed using RIPA lysis and extraction buffer (Thermo Fisher Scientific, 89901) containing a protease inhibitor cocktail. The lysates were mixed with 5× SDS PAGE sample loading buffer and boiled for 10 min to denature the proteins. The samples were separated by SDS-PAGE and transferred onto NC membranes (GE Healthcare, 10600001). The membrane was incubated with the indicated primary and secondary antibodies. Finally, protein signals were detected using LumiQ ECL electrochemiluminescence (Share-bio, SB-WB012).

### RNA extraction, reverse-transcriptase quantitative PCR, and tagged-primer strand-specific Qpcr

According to the experimental protocol, total RNA was extracted using either the FastPure Viral DNA/RNA Mini Kit (Vazyme Biotech, RC311) or the FastPure Cell/Tissue Total RNA Isolation Kit (Vazyme Biotech, RC112). The RNA was reverse-transcribed into cDNA using the HiScript III RT SuperMix for qPCR (+gDNA wiper) (Vazyme Biotech, R323-01) or the HiScript II 1st Strand cDNA Synthesis Kit (+gDNA wiper) (Vazyme Biotech, R212-01) for qPCR analysis with sequence-specific primers. RT-qPCR was performed following the instructions of the ChamQ Universal SYBR qPCR Master Mix (Vazyme Biotech, q711-03). The primer sequences are provided in Table S4. The relative RNA expression levels were calculated using the 2^–ΔΔCT^ method, with GAPDH serving as the internal reference.

### RNA immunoprecipitation and specific target protein-focused *in vitro*-transcribed RNA immunoprecipitation

For the RIP experiments, about 5–20 × 10^6^ PEDV-infected cells were used per condition (63). At the indicated time points, the medium was removed, and the cells were washed once with prechilled PBS. Then, the cells were lysed with polysome lysis buffer(64) (prepared with 10 mM HEPES, 100 mM KCl, 5 mM MgCl_2_, 0.5% NP40, 1 mM DTT, 100 U/mL RNase, 1% protease inhibitor cocktail, 400 µM vanadyl ribonucleoside complexes (VRCs), and DNase-RNase-free distilled water), scraped in lysis buffer, incubated on ice for 15 min, and the RNP lysates were collected and stored at −80 °C. For the STPIVT-RIP experiments, the HEK 293T cells were lysed on ice for 30 min in Cell Lysis Buffer II (Thermo Fisher Scientific, FNN0021) containing a protease inhibitor cocktail, and the lysate was mixed at a 1:1 ratio.

For the RIP experiments, the Dynabeads Protein G beads and NT2 buffer (64) (prepared with 50 mM Tris-HCl, 150 mM NaCl, 1 mM MgCl_2_, 0.05% NP40, and DNase-RNase-free distilled water) containing 5% bovine serum albumin (BSA) were mixed at a 5:1 ratio, and the mixture was incubated at 4 °C for at least 1 h. After incubation, the anti-Flag or anti-IgG antibodies were added to the beads, which were subsequently rotated and incubated overnight at 4 °C. After the supernatant was removed, the antibody-conjugated beads were immediately washed 4–5 times with ice-cold NT2 buffer. The RNP lysate was added to the antibody-conjugated magnetic beads and rotated for incubation at 4 °C for 4 h. For the STPIVT-RIP experiments, the mixed proteins and *in vitro*-transcribed RNA were added to antibody-conjugated magnetic beads for 4 h at 4 °C. The beads were washed 4–5 times with ice-cold NT2 buffer, after which the beads were resuspended in NT2 buffer as the IP group or IgG group. The RNA from the IP and IgG groups was extracted with TRIzol for RT-qPCR, following the previous procedure.

### RIP-seq assay

The RNA from the IP and IgG groups was extracted with TRIzol. Total RNA quality was assessed via concentration and purity using a NanoDrop 2000 instrument (Thermo Fisher Scientific, Waltham, Massachusetts, USA), whereas its integrity was verified via RNA-specific agarose electrophoresis. Library preparation was performed using NEB Next Multiplex Small RNA Library Prep Set for Illumina (NEB, E7560S). 3′ and 5′ adapters were ligated using ligase, followed by reverse transcription with Superscript II to synthesize double-stranded cDNA. PCR amplification enriched DNA fragments, which were then size-selected using 15% PAGE gel to obtain target-size products. Final libraries underwent quality control with Agilent High Sensitivity DNA Kit (Agilent, 5067-4626). Total library concentration was measured using Picogreen (Quant-iT PicoGreen dsDNA Assay Kit (Thermo Fisher Scientific, P7589), with effective concentration quantified by qPCR. Multiplexed DNA libraries were normalized and pooled at equal volumes. After stepwise dilution and quantification, pooled libraries were sequenced on Illumina platforms using PE150 mode.

### Confocal immunofluorescence assay

HeLa or LLC-PK1 cells were cultured in six-well plates containing climbing slices. After the cells were fixed with a fixation solution (4% paraformaldehyde, 4% sucrose, pH 7.4), the cell samples were permeabilized with 0.1% Triton X-100, followed by 60 min of incubation with 3% BSA-diluted primary and secondary antibodies at 37 °C (65). Finally, the cells were observed by confocal immunofluorescence microscopy (Carl Zeiss, Germany).

### RNA-scope

LLC-PK1 cells were cultured in 24-well plates containing climbing slices and inoculated with PEDV at an MOI of 200. Next, 4 h post-infection, the cell samples were pretreated with RNAscope H_2_0_2_ and Protease Reagents (ACD, 3322381) following the manufacturer’s instructions. The RNA obtained was stained using the RNAscope Multiplex Fluorescent Reagent Kit (ACD, 323110) and RNAscope Wash Buffer Reagents (ACD, 310091) following the manufacturer’s protocol. The climbing slices were mounted on parafilm-wrapped glass slides. The probes and fluorophores included RNAscope Probe-V-PEDV-N (ACD, 510961) and Opal 570 (ACD, RE202170).

To combine immunostaining with RNA staining, the cells were permeabilized with 0.1% Triton X-100 after RNA staining was complete. Next, the cells were incubated with 3% BSA-diluted primary and secondary antibodies at 37 °C for 60 min. The cells were observed using a laser scanning confocal microscope (Carl Zeiss, Germany).

### Coimmunoprecipitation assay

The cells were lysed on ice for 30 min using Cell Lysis Buffer II containing a protease inhibitor cocktail. The supernatant was collected or mixed at a 1:1:1 ratio for Co-IP. Next, the protein samples were bound to Dynabeads protein G by incubating the beads with a Flag antibody. Next, 0.02% PBST was applied to rinse the Dynabeads. The last step involved protein elution with 50 mM glycine buffer (pH 2.8), followed by detection via western blotting.

### GST affinity-isolation assay

Following the experimental protocol, the prokaryotic plasmid of the target gene was expressed in BL21 competent cells (Vazyme Biotech, C504-03). The bacterial culture mixture was collected by centrifugation at 4 °C and 12,000 rpm for 1 min, and protein-protein interactions were performed using GST protein interaction pull-down kits (Thermo Fisher Scientific, 21516) following the manufacturer’s instructions.

### Transmission electron microscopy

LLC-PK1 cells were cultured in T75 flasks with post-transfection densities exceeding 90% for PEDV infection (200 MOI). Next, 4 h post-infection, the cells were fixed with precooled electron microscopy fixative following the manufacturer’s instructions and stored at 4 °C. Samples were rinsed with 0.1M phosphate buffer (pH 7.4), then fixed with 1% osmium tetroxide (in 0.1M phosphate buffer, pH 7.4) at room temperature. Gradient dehydration was followed using 30%, 50%, 70%, 80%, 85%, 90%, and 100% ethanol (twice) sequentially. Dehydrated samples underwent progressive infiltration in acetone:epoxy resin mixtures (2:1 and 1:1 ratios), followed by pure epoxy resin, each step lasting 8–12 h at 37 ℃. Samples were embedded in embedding molds with epoxy resin and polymerized at 60 ℃ for 48 h. Polymerized blocks were sectioned for dual staining with 2% uranyl acetate aqueous solution and lead citrate and finally observed by electron microscopy.

### Metabolic labeling of heavy-chain amino acids

LLC-PK1 cells were cultivated in MEM supplemented with 10% heat-inactivated FBS, 84 mg/L ^13^C6-L-arginine, and 146 mg/L ^13^C6-L-lysine for 7 days. The cells were transfected with Flag-RNMT or Flag plasmids and infected with PEDV. The viral inoculum was prepared with MEM containing 3 µg/mL trypsin (0.25%), 100 U/mL penicillin-streptomycin, 84 mg/L ^13^C6-L-arginine, and 146 mg/L ^13^C6-L-lysine. At 1 h post-infection, the cells were lysed on ice for 30 min in Cell Lysis Buffer II containing a protease inhibitor cocktail. The protein samples were incubated with the PEDV N antibody binding beads for the Co-IP assay. The proteins were eluted with 80 µL of SDT buffer for mass spectrometry analysis.

### RIP-seq data analysis

Data quality control: The samples were sequenced using the sequencing platform, generating image files that were converted by the platform’s built-in software to produce raw data in FASTQ format (raw data), referred to as off-machine data. In-house-developed scripts were used to perform adapter removal from the off-machine data, followed by quality trimming based on sequence quality. A sliding window approach (5-base window) was used to scan raw sequences, where sequences were truncated and discarded from the window starting position when the average sequencing quality score within the window dropped below 20. Clean reads (total reads) with lengths ranging from 18 to 36 nt were counted. Identical sequences in individual samples were deduplicated, and their sequence abundances were quantified, generating unique reads for subsequent analysis.

Sequence alignment to the reference genome: Genome alignment analysis was performed using the miRDeep2 software (66) (Mackowiak SD, 2011) to map unique reads against the swine reference genome sequence. Additionally, the Bowtie v2.4.1 software (67) was used to align the clean reads to the PEDV reference genome sequence, generating alignment files in the BAM format. The read coverage peak profiles were visualized using the IGV software (68).

### Protein identification and quantitative analysis

The raw files from mass spectrometry tests were analyzed using the MaxQuant v2.1.1.1 software (69) with the following parameter settings: the main search ppm was set to 6 ppm, and the maximum number of missed cleavages was 2. Trypsin digestion was selected with deisotopic processing enabled (De-Isotopic: TRUE). Fixed modifications included carbamidomethyl (C), whereas variable modifications included oxidation (M). Databases were searched against the actual database name with a reverse decoy database pattern for false-positive control (decoy database pattern: reverse). For label-free quantification (LFQ), the parameters used were an LFQ min ratio count of 1 and match between runs enabled (Match between runs: 2 min). The peptide mass tolerance was set at ±20 ppm. Strict false discovery rate (FDR) thresholds were applied (Peptide FDR ≤ 0.01, Protein FDR ≤ 0.01). The final results included protein identification and quantitative data analysis.

### Protein-protein interaction network

The UniProt IDs of proteins identified in each sample were submitted to the UniProt database to retrieve the corresponding protein sequences. Then, the sequences were analyzed for protein-protein interactions using the STRING software (70) with default parameters. The protein interaction results were imported into the R environment, and the interaction network was visualized using tidygraph v1.3.1 and scatterpie v0.2.4, with the color intensity representing Log_10_^(LFQ intensity + 10)^.

### Statistical analysis

The data were analyzed using the GraphPad Prism 5.0 software and presented as the mean ± standard deviation. Significance was determined by two-way analysis of variance (ANOVA) or one-way ANOVA. ns, *P* > 0.05, not significant; *P ≤ 0.05, significant; **P ≤ 0.01, very significant; ***P ≤ 0.001, highly significant.

## Data Availability

All relevant data are within the manuscript and its Supporting Information files. The mass spectrometry proteomics data have been deposited to the ProteomeXchange Consortium via the PRIDE partner repository with the data set identifier PXD067906. The RNA-seq data are available from NCBI SRA database under the accession number PRJNA1313363.

## SUPPLEMENTAL MATERIAL

Supplemental information mbio.02717-25-s0001.docx Fig. S1 to S8; Table S1 to S4 captions.

Table S1 mbio.02717-25-s0002.xlsx Quantitative mass spectrometry data for coronavirus RNA interactomes.

Table S2 mbio.02717-25-s0003.xlsx Analysis of coronavirus RNA interactomes.

Table S3 mbio.02717-25-s0004.xlsx DDX5 protein-protein interactome in PEDV-infected cells.

Table S4 mbio.02717-25-s0005.xlsx All primers used.

ASM does not own the copyrights to Supplemental Material that may be linked to, or accessed through, an article. The authors have granted ASM a non-exclusive, world-wide license to publish the Supplemental Material files. Please contact the corresponding author directly for reuse.
