# Supplementary material for: Nucleocapsid protein captures DDX5 and RNMT facilitating viral RNA synthesis and viral protein translation for coronavirus replication
Source: mBio. 2026 Jan 26;17(3):e02717-25. doi: 10.1128/mbio.02717-25 (PMC12977548; doi:10.1128/mbio.02717-25)
Supplement: Supplemental information — Fig. S1 to S8; Table S1 to S4 captions. [file mbio.02717-25-s0001.docx]

**Supplemental Information**

**Nucleocapsid protein captures DDX5 and RNMT facilitating viral RNA synthesis and viral protein translation for coronavirus replication**

Yuchang Liu^a*^, Ning Kong^a,b*^, Xinyu Yang^a*^, Wenzhen Qin^a^, Yahe Wang^a^, Chen Wang^a^, He Sun^a^, Jiarui Wang^a^, Ao Gao^a^, Dongfang Zheng^a^, Wu Tong^a^, Hai Yu^a^, Hao Zheng^a^, Guangzhi Tong^a^, Tongling Shan^a,b#^

**Supplemental figures**

**
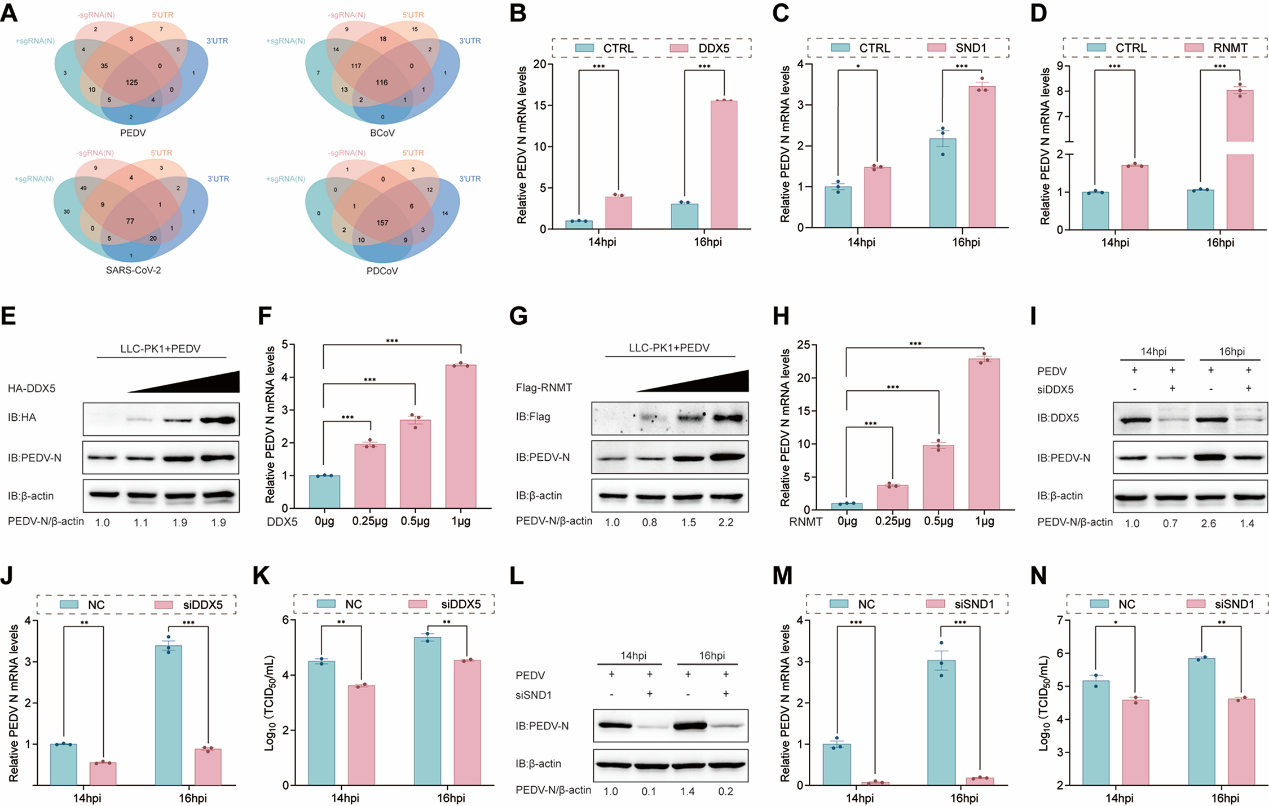
**

**Fig S1** Analysis of the proteins that interact with the RNA of each coronavirus and the host proteins DDX5, SND1, and RNMT to promote viral replication. (**A**) Venn diagrams were used to compare the protein interactomes of four bait RNAs (±sgRNA of N, 5'-UTR, and 3'-UTR) from PEDV, BCoV, SARS-CoV-2, and PDCoV. (**B-D**) RT-qPCR analyses of PEDV replication in LLC-PK1 cells transfected with HA-DDX5, Flag-SND1, or Flag-RNMT plasmids. The cells were infected with PEDV (MOI = 1) and harvested at the indicated time points after viral infection. LLC-PK1 cells were transfected with HA or Flag plasmids as a control (CTRL). p values were determined by two-way ANOVA. Data are presented as mean ± SD from three replicate samples. (**E-H**) Western blotting and RT-qPCR analyses of PEDV replication in PEDV-infected LLC-PK1 cells transfected with HA-DDX5 or Flag-RNMT plasmids at different concentrations; β-actin served as a control. p values were determined by one-way ANOVA. Data are presented as mean ± SD from three replicate samples. (**I-N**) Western blotting, RT-qPCR, and TCID_50_ analyses of PEDV replication in siDDX5-, siSND1-, or NC-transfected LLC-PK1 cells infected with PEDV (MOI = 1); β-actin served as a control. p values were determined by two-way ANOVA. Data are presented as mean ± SD from three replicate samples. ***p < 0.001, **p < 0.01, *p < 0.05, ns = no statistical significance.


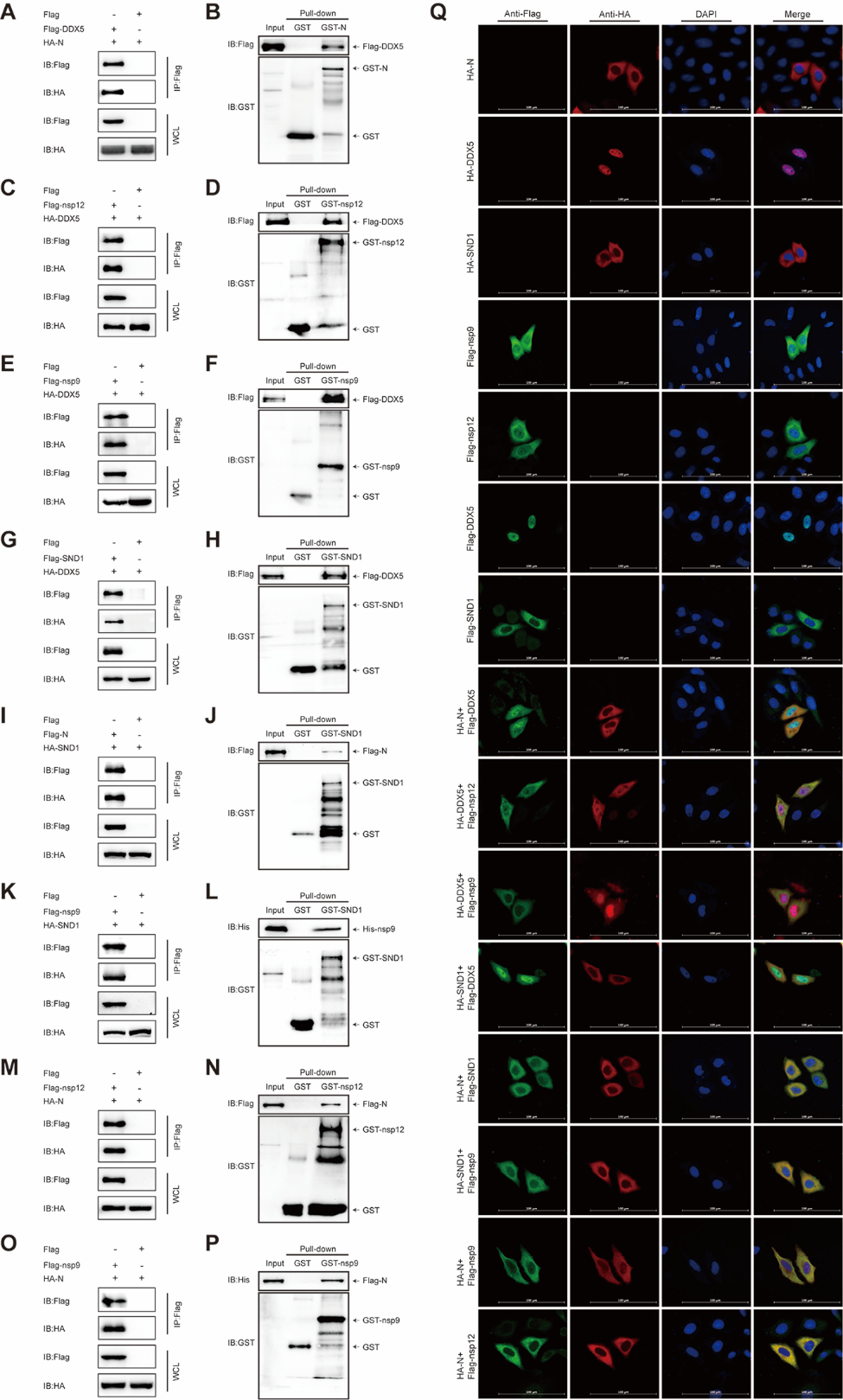


**Fig S2** DDX5 and SND1 interact with RTC components involved in viral RNA biogenesis. (**A**) Flag-DDX5 and HA-N plasmids were co-transfected into HEK 293T cells, and protein interactions were detected by Co-IP. (**B**) Flag-DDX5 and GST-N recombinant proteins were expressed in BL21 *E. coli* strains, and GST pull-down assays were performed to investigate the protein interactions between those two proteins. (**C-H**) The interactions between DDX5 and nsp12, nsp9, or SND1 were investigated via Co-IP and GST pull-down assays. (**I-L**) The interactions between SND1 and N or nsp9 were investigated via Co-IP and GST pull-down assays. (**M-P**) The interactions between N and nsp12 or nsp9 were investigated via Co-IP and GST pull-down assays. (**Q**) Immunofluorescence staining of nsp12, DDX5, N, nsp9, and SND1 in HeLa cells was performed by confocal immunofluorescence microscopy, with a scale bar of 100 µm.


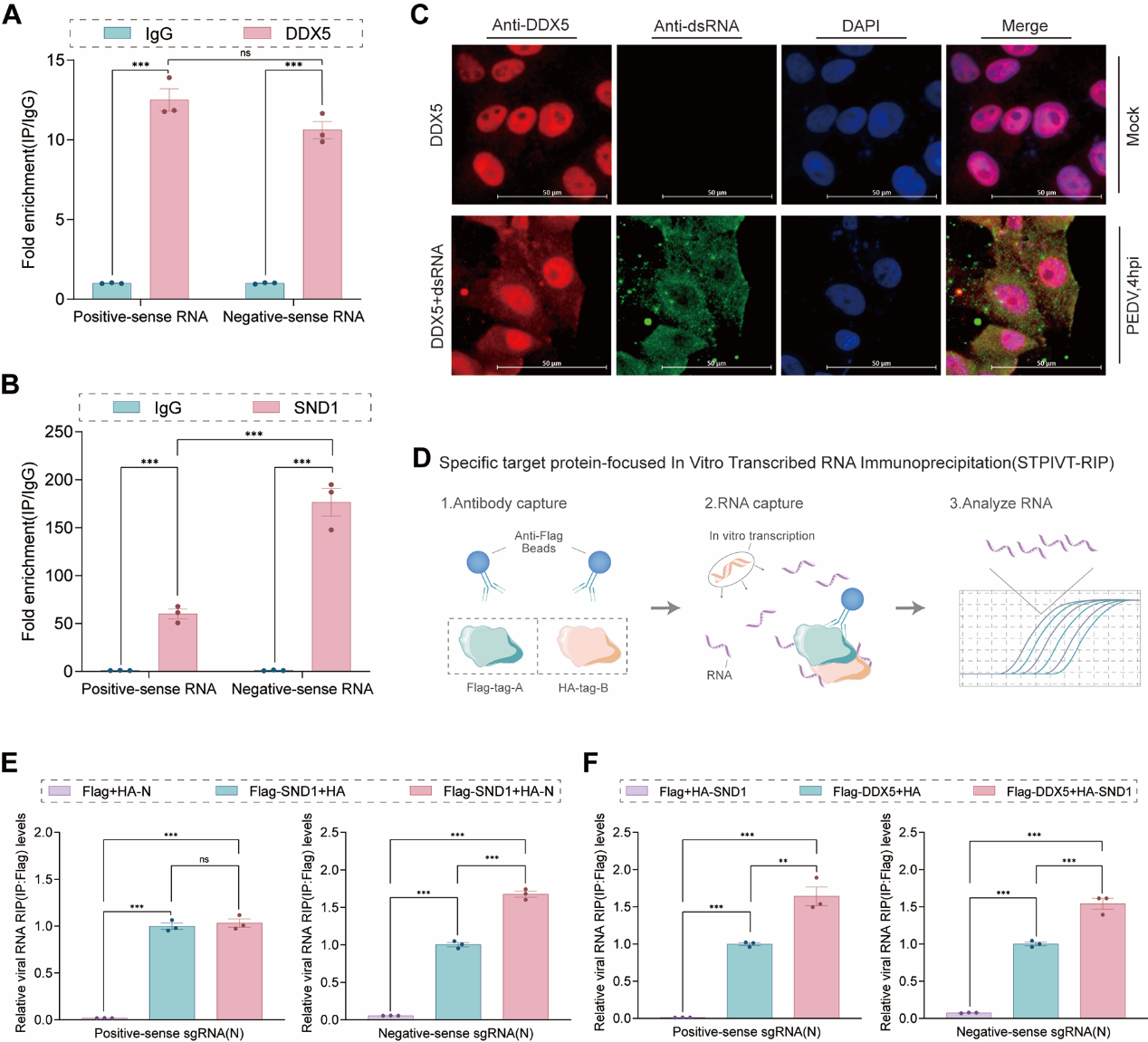


**Fig S3** Analysis of the effects of DDX5 and SND1 binding to positive-sense and negative-sense PEDV RNA. (**A**) RIP analysis of PEDV RNA was performed using an anti-Flag antibody or IgG in Flag-DDX5 plasmid-transfected LLC-PK1 cells infected with PEDV (20 hpi, MOI = 1). The data are presented as fold enrichment, with the IP-IgG group used as a negative control. Differential analysis was performed between the IP-Flag groups for positive-sense and negative-sense RNA. p-values determined by one-way ANOVA. Data are presented as mean ± SD from three replicate samples. (**B**) RIP analysis of PEDV RNA was performed using an anti-Flag antibody or an IgG antibody in Flag-SND1 plasmid-transfected LLC-PK1 cells infected with PEDV (4 hpi). The data are presented as fold enrichment, with the IP-IgG group serving as a negative control. Differential analysis was performed for SND1-binding positive-sense RNA and negative-sense RNA. p-values determined by one-way ANOVA. Data are presented as mean ± SD from three replicate samples. (**C**) Immunofluorescence staining of DDX5 with dsRNA in PEDV-infected LLC-PK1 cells at 4 hpi was observed by confocal immunofluorescence microscopy. Scale bar: 50 µm. (**D**) Schematic illustration of the specific target protein-focused in vitro-transcribed RNA immunoprecipitation (STPIVT-RIP) workflow enabling specific discrimination between viral positive-sense and negative-sense RNA levels. (**E**) After the transfection of HEK 293T cells with plasmids encoding Flag-SND1, Flag, HA-N, or HA, combinations of Flag+HA-N, Flag-SND1+HA, and Flag-SND1+HA-N (1:1 ratio) were analyzed by STPIVT-RIP with in vitro-transcribed positive-sense sgRNA (N) or negative-sense sgRNA (N). For quantitative analysis, Flag+HA-N was used as a blank control relative to Flag-SND1+HA. p-values were determined by one-way ANOVA. Data are presented as mean ± SD from three replicate samples. (**F**) After the transfection of HEK 293T cells with plasmids encoding Flag-DDX5, Flag, HA-SND1, or HA, combinations of Flag+ HA-SND1, Flag-DDX5+HA, and Flag-DDX5+ HA-SND1 (1:1 ratio) were analyzed by STPIVT-RIP with in vitro-transcribed positive-sense sgRNA (N) or negative-sense sgRNA (N). p-values were determined by one-way ANOVA. Data are presented as mean ± SD from three replicate samples. ***p < 0.001, **p < 0.01, *p < 0.05, ns = not significant.


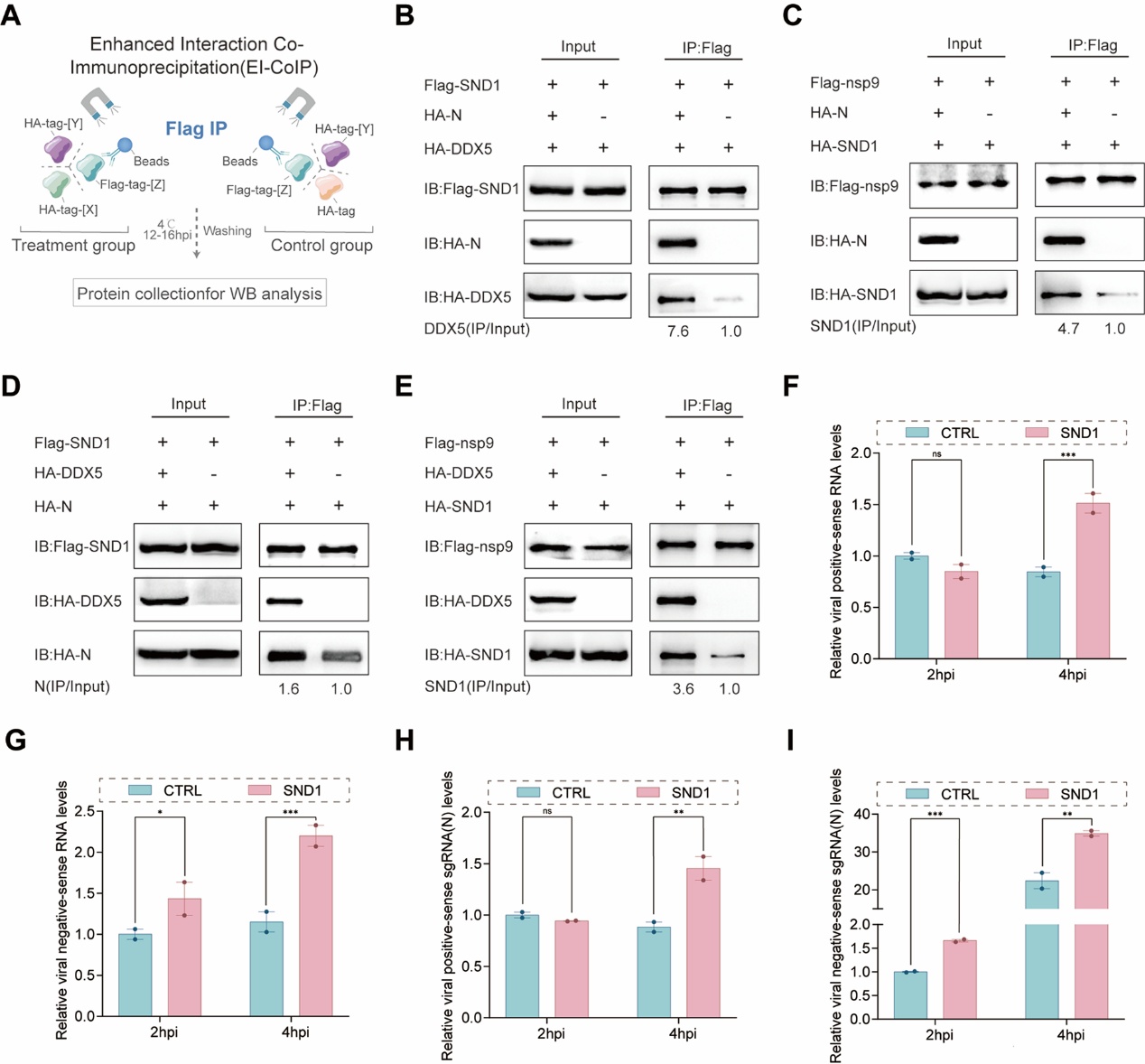


**Fig S4** DDX5 efficiently promotes the recruitment of core components involved in RTC. (**A**) Schematic workflow of Co-IP analysis of the influence of a protein on the interactions between the other two proteins. (**B and C**) Co-IP analysis of the effects of N on the interactions between SND1 and DDX5 or between nsp9 and SND1. Lysates from HEK 293T cells transfected with Flag-SND1, Flag-nsp9, HA-DDX5, HA-SND1, HA-N, or HA plasmids were mixed at a 1:1:1 ratio. (**D and E**) Co-IP analysis of the effect of DDX5 on the interaction between SND1 and N, nsp9, and SND1. Lysates from HEK 293T cells transfected with Flag-SND1, Flag-nsp9, HA-SND1, HA-DDX5, HA-N, or HA plasmids were mixed at a 1:1:1 ratio. (**F-I**) TP-SS qPCR analysis of PEDV positive-sense RNA, negative-sense RNA, positive-sense sgRNA (N), and negative-sense sgRNA (N) levels in Flag- or Flag-SND1-transfected LLC-PK1-infected cells affected by PEDV (MOI = 200). The data were normalized to GAPDH as an internal reference and quantified relative to Flag at 2 hpi. p-values determined by two-way ANOVA. Data are presented as mean ± SD from two replicate samples. ***p < 0.001, **p < 0.01, *p < 0.05, ns = not significant.


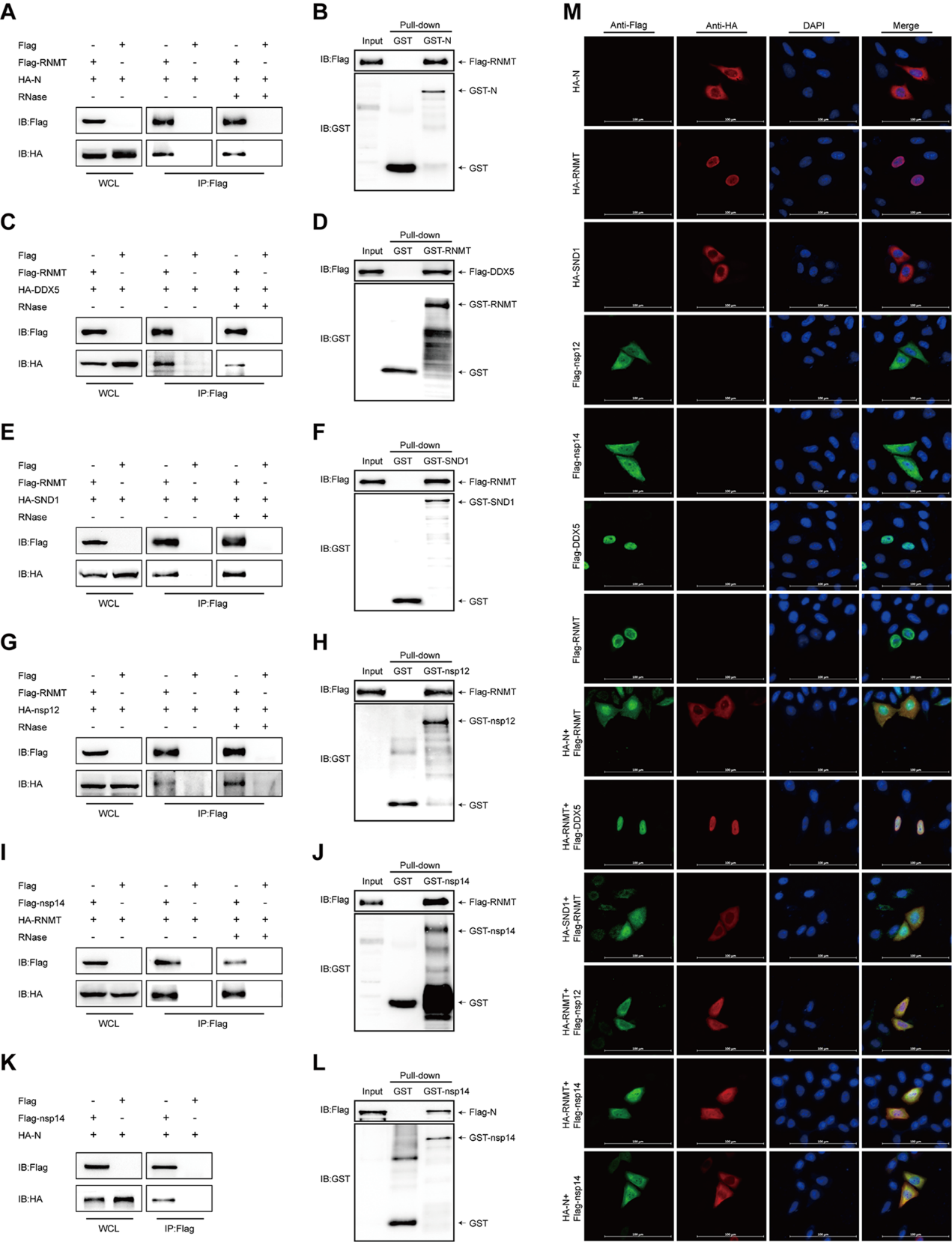


**Fig S5** Analysis of the interaction of RNMT and RTC components involved in viral RNA biogenesis. (**A**) Flag-RNMT and HA-N plasmids were co-transfected into HEK 293T cells, and protein interactions were detected by Co-IP. (**B**) Flag-RNMT and GST-N recombinant proteins were expressed in BL21 *E. coli* strains, and GST pull-down assays were performed to investigate the protein interactions between those two proteins. (**C-J**) RNMT interactions with DDX5, SND1, nsp12, and nsp14 were investigated via co-IP and GST pull-down assays. (**K and L**) PEDV N and nsp14 protein interactions were investigated by Co-IP and GST pull-down assays. (**M**) Immunofluorescence staining of RNMT, DDX5, SND1, N, nsp12, and nsp14 in HeLa cells was performed via confocal immunofluorescence microscopy, with a scale bar of 100 µm.


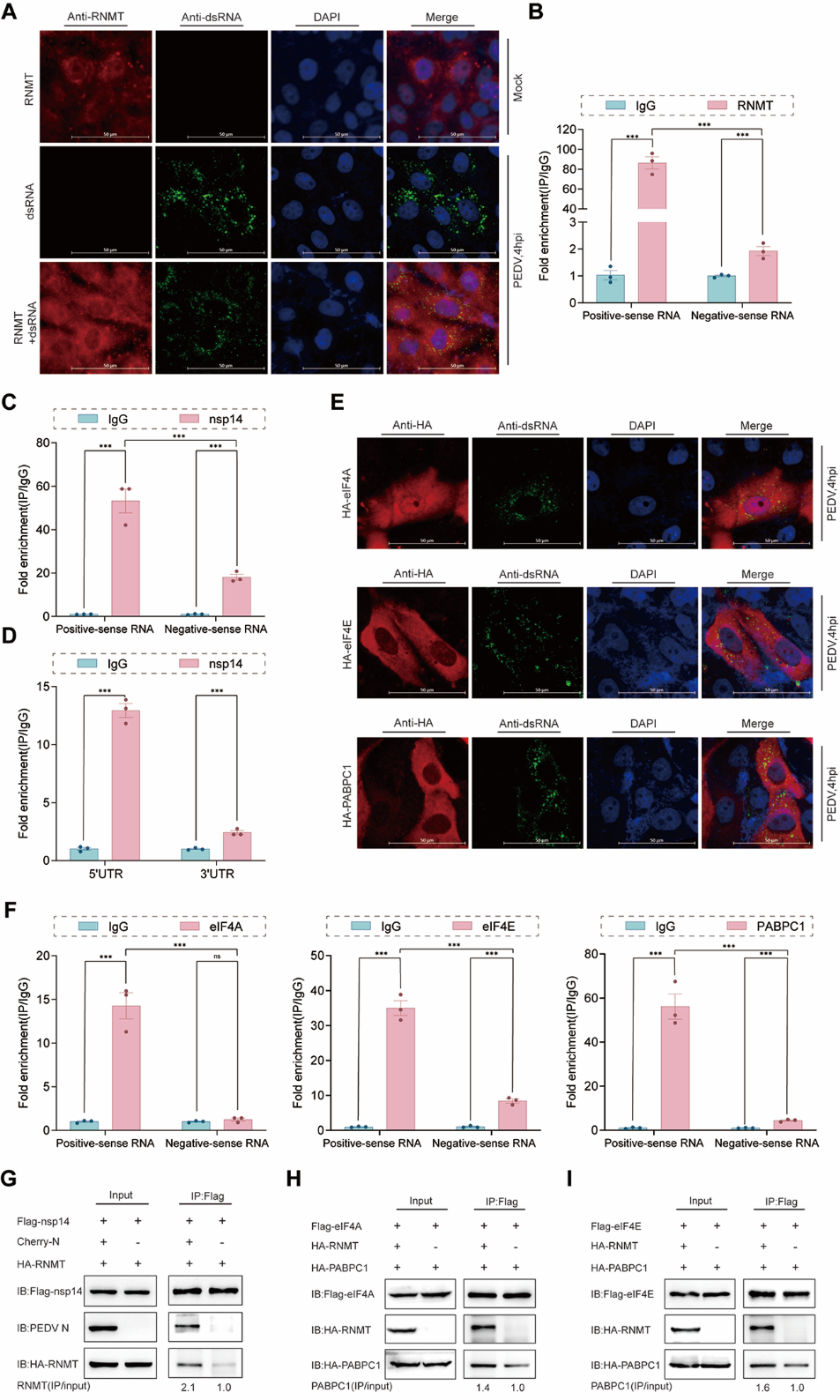


**Fig S6** Analysis of RNMT, viral nsp14, eIF4A, eIF4E and PABPC1 binding viral RNA patterns. (**A**) Immunofluorescence staining of RNMT and dsRNA in PEDV-infected LLC-PK1 cells at 4 hpi was performed via confocal microscopy. Scale bar: 50 µm. (**B**) RIP analysis of PEDV RNA using an anti-Flag antibody or IgG in Flag-RNMT plasmid-transfected LLC-PK1 cells infected with PEDV (4 hpi). Differential analysis was performed for positive-sense RNA and negative-sense RNA bound to RNMT. p-values determined by one-way ANOVA. Data are presented as mean ± SD from three replicate samples. (**C and D**) RIP analysis of PEDV RNA using an anti-Flag antibody or IgG in Flag-nsp14 plasmid-transfected LLC-PK1 cells infected with PEDV (4 hpi). Differential analysis was performed for nsp14 binding to positive-sense RNA, negative-sense RNA, the 5'UTR, and the 3'UTR of positive-sense RNA. p-values determined by one-way ANOVA. Data are presented as mean ± SD from three replicate samples. (**E**) Immunofluorescence staining of HA-eIF4A/eIF4E/PABPC1 and dsRNA in PEDV-infected and HA-eIF4A/eIF4E/PABPC1 plasmid-transfected LLC-PK1 cells at 4 hpi was performed via confocal microscopy. Scale bar: 50 µm. (**F**) RIP analysis of PEDV RNA using an anti-Flag antibody or IgG in Flag-eIF4A/eIF4E/PABPC1 plasmid-transfected LLC-PK1 cells infected with PEDV (4 hpi). Differential analysis was performed for eIF4A/eIF4E/PABPC1 binding positive-sense RNA and negative-sense RNA of positive-sense RNA. p-values determined by one-way ANOVA. Data are presented as mean ± SD from three replicate samples. (**G**) Co-IP analysis of the effect of N on the interaction between nsp14 and RNMT. Lysates from HEK 293T cells transfected with Flag-nsp14, HA-RNMT, or HA plasmids were mixed at a 1:1:1 ratio. (**H and I**) Co-IP analysis of the effects of RNMT on the interactions between eIF4A and PABPC1 and between eIF4E and PABPC1. Lysates from HEK 293T cells transfected with Flag-eIF4A, Flag-eIF4E, HA-RNMT, HA-PABPC1, or HA plasmids were mixed at a 1:1:1 ratio. ***p < 0.001, **p < 0.01, *p < 0.05, ns = not significant.


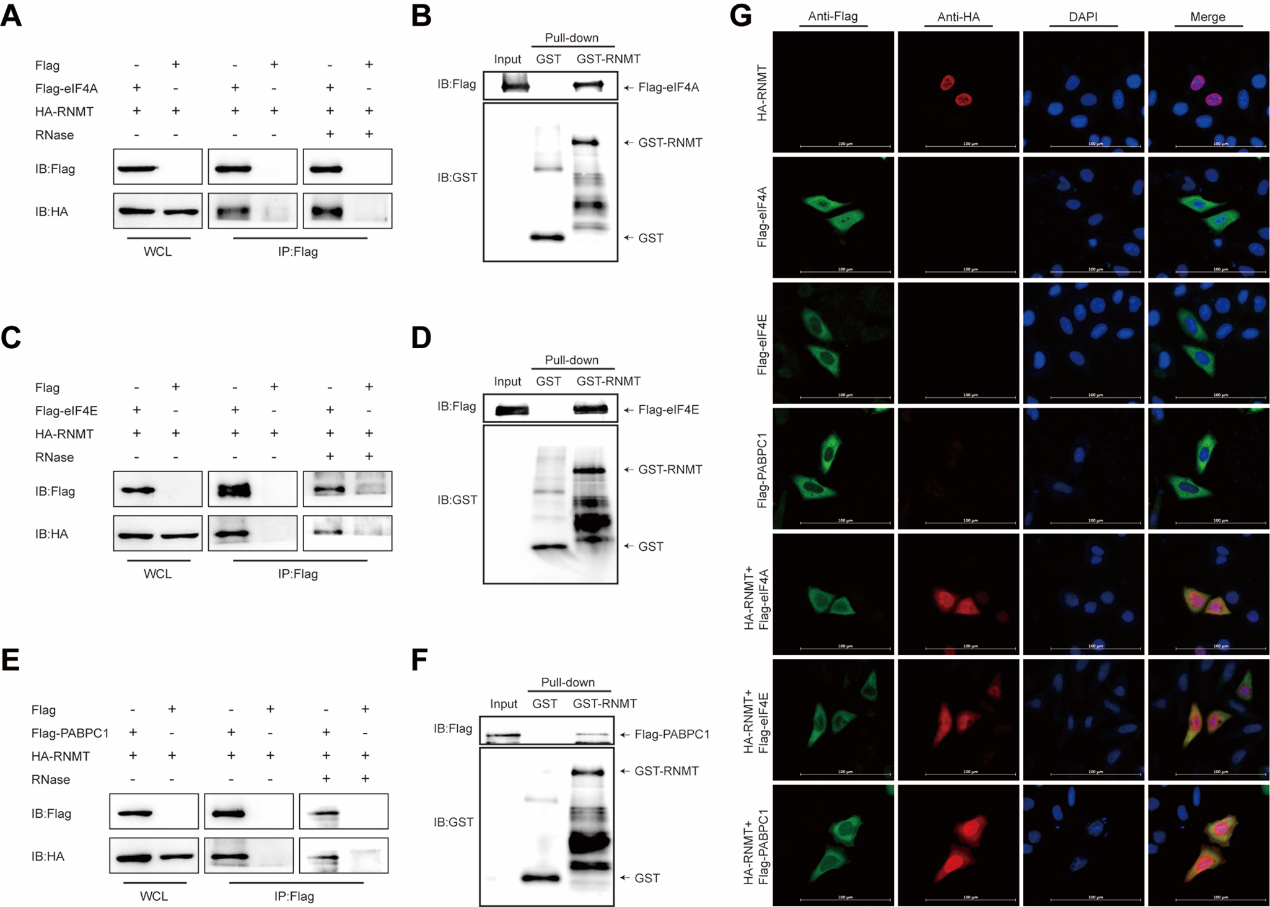


**Fig S7** Analysis of the interaction between RNMT and protein translation components. (**A**) Flag- eIF4A and HA-RNMT plasmids were co-transfected into HEK 293T cells, and protein interactions were detected by Co-IP. (**B**) Flag- eIF4A and GST-RNMT recombinant proteins were expressed in BL21 *E. coli* strains, and GST pull-down assays were performed to investigate the protein interactions between those two proteins. (**C-F**) RNMT interacted with eIF4E, and PABPC1 was investigated by co-IP and GST pull-down assays. (**G**) Immunofluorescence staining of RNMT, eIF4A, eIF4E, and PABPC1 in HeLa cells was performed via confocal immunofluorescence microscopy, with a scale bar of 100 µm.


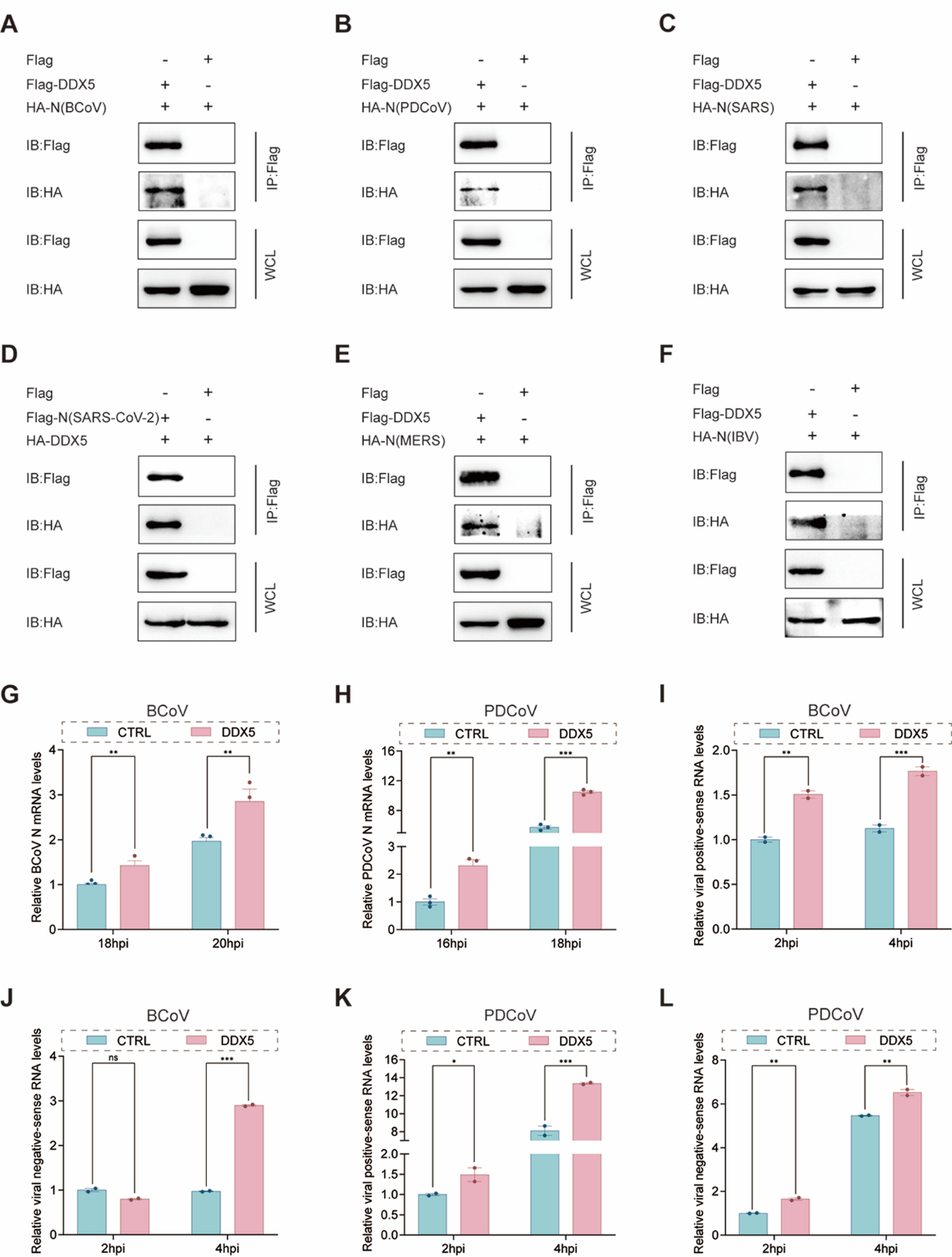


**Fig S8** DDX5 interacts with the viral N protein and viral RNA to promote coronavirus replication. (**A-F**) After co-transfecting HEK 293T cells with plasmids encoding DDX5 and N (BCoV, PDCoV, SARS, SARS-CoV-2, MERS, and IBV), protein interactions were detected by Co-IP. (**G**) RT-qPCR analysis of BCoV RNA levels in the supernatants of Vero cells transfected with HA or HA-DDX5 and infected with BCoV (MOI = 1). Quantification was performed relative to the HA group (CTRL) at 18 hpi. p-values were determined by two-way ANOVA. Data are presented as mean ± SD from three replicate samples. (**H**) RT-qPCR analysis of PDCoV RNA levels in supernatants of LLC-PK1 cells transfected with HA or HA-DDX5 and infected with PDCoV (MOI = 1). Quantification was performed relative to the HA group (CTRL) at 16 hpi. p-values determined by two-way ANOVA. Data are presented as mean ± SD from three replicate samples. (**I and J**) TP-SS qPCR analysis of BCoV positive-sense RNA and negative-sense RNA levels in HA- or HA-DDX5-transfected Vero cells infected with BCoV (MOI = 200). GAPDH served as an internal control, with quantification relative to the HA group (CTRL) after infection for 2 h. p-values determined by two-way ANOVA. Data are presented as mean ± SD from two replicate samples. (**K and L**) TP-SS qPCR analysis of PDCoV positive-sense RNA and negative-sense RNA levels in HA- or HA-DDX5-transfected LLC-PK1 cells infected with PDCoV (MOI = 200). p-values determined by two-way ANOVA. Data are presented as mean ± SD from two replicate samples. ***p < 0.001, **p < 0.01, *p < 0.05, ns = not significant.

**Supplemental tables**

**Table S1** Quantitative mass spectrometry data for coronavirus RNA interactomes.

**Table S2** Analysis of coronavirus RNA interactomes.

**Table S3** DDX5 protein-protein interactome in PEDV infected cells.

**Table S4** All primers used for this manuscript.
